# A Novel MiRNA-Based Predictive Model for Biochemical Failure Following Post-Prostatectomy Salvage Radiation Therapy

**DOI:** 10.1371/journal.pone.0118745

**Published:** 2015-03-11

**Authors:** Erica Hlavin Bell, Simon Kirste, Jessica L. Fleming, Petra Stegmaier, Vanessa Drendel, Xiaokui Mo, Stella Ling, Denise Fabian, Isabel Manring, Cordula A. Jilg, Wolfgang Schultze-Seemann, Maureen McNulty, Debra L. Zynger, Douglas Martin, Julia White, Martin Werner, Anca L. Grosu, Arnab Chakravarti

**Affiliations:** 1 Department of Radiation Oncology, Arthur G. James Hospital/ Ohio State Comprehensive Cancer Center, Columbus, Ohio, United States of America; 2 Department of Radiation Oncology, University Medical Center Freiburg, Freiburg, Germany; 3 Department of Pathology, University Medical Center Freiburg, Freiburg, Germany; 4 Center for Biostatistics, The Ohio State University Wexner Medical Center, Columbus, Ohio, United States of America; 5 Department of Urology, University Medical Center Freiburg, Freiburg, Germany; 6 The Department of Pathology, The Ohio State University Wexner Medical Center, Columbus, Ohio, United States of America; Innsbruck Medical University, AUSTRIA

## Abstract

**Purpose:**

To develop a microRNA (miRNA)-based predictive model for prostate cancer patients of 1) time to biochemical recurrence after radical prostatectomy and 2) biochemical recurrence after salvage radiation therapy following documented biochemical disease progression post-radical prostatectomy.

**Methods:**

Forty three patients who had undergone salvage radiation therapy following biochemical failure after radical prostatectomy with greater than 4 years of follow-up data were identified. Formalin-fixed, paraffin-embedded tissue blocks were collected for all patients and total RNA was isolated from 1mm cores enriched for tumor (>70%). Eight hundred miRNAs were analyzed simultaneously using the nCounter human miRNA v2 assay (NanoString Technologies; Seattle, WA). Univariate and multivariate Cox proportion hazards regression models as well as receiver operating characteristics were used to identify statistically significant miRNAs that were predictive of biochemical recurrence.

**Results:**

Eighty eight miRNAs were identified to be significantly (p<0.05) associated with biochemical failure post-prostatectomy by multivariate analysis and clustered into two groups that correlated with early (≤ 36 months) versus late recurrence (>36 months). Nine miRNAs were identified to be significantly (p<0.05) associated by multivariate analysis with biochemical failure after salvage radiation therapy. A new predictive model for biochemical recurrence after salvage radiation therapy was developed; this model consisted of miR-4516 and miR-601 together with, Gleason score, and lymph node status. The area under the ROC curve (AUC) was improved to 0.83 compared to that of 0.66 for Gleason score and lymph node status alone.

**Conclusion:**

miRNA signatures can distinguish patients who fail soon after radical prostatectomy versus late failures, giving insight into which patients may need adjuvant therapy. Notably, two novel miRNAs (miR-4516 and miR-601) were identified that significantly improve prediction of biochemical failure post-salvage radiation therapy compared to clinico-histopathological factors, supporting the use of miRNAs within clinically used predictive models. Both findings warrant further validation studies.

## Introduction

Prostate cancer (PCa) is one of the most common cancers worldwide and the most common cancer in men; however, treatment strategies remain highly controversial. Radical prostatectomy (RP) remains one of the more widely-used treatment options for men with early-stage PCa. Long-term data indicate that 30–40% of these patients experience biochemical failure after RP requiring salvage radiation therapy (RT); however, other studies have shown significantly different incidences due to different clinical prognostic characteristics of tumors [1–3]. The key clinical questions that are the focus of the current study are the identification of: 1) microRNAs (miRNAs) that predict biochemical recurrence after RP; 2) miRNAs that predict for biochemical recurrence after salvage radiation following failure after RP; and 3) miRNAs that can improve prediction of biochemical recurrence in combination with currently used clinico-histopathological factors, such as prostate-specific antigen (PSA), pathologic tumor (pT) and lymph node (pN) classification, resection status, and Gleason score. Multiple nomograms and classification models have been derived utilizing traditional clinico-histopathological parameters (CAPRA score [4–6], Partin table [7,8], D’Amico classification [9], and the three Stephenson Nomograms [10–12]) in an effort to establish prognosis, but these all have limitations in the context of salvage RT. Genetic markers to help guide decision making processes are also being developed for PCa as they have been for breast cancer, such as OncotypeDX [13] and a gene expression-based genomic classifier [14,15]. The goal of this study was to develop a miRNA signature that can add information to the existing clinical models and thereby help guide treatment decisions.

miRNAs are small (∼22 nucleotides), non-coding RNAs that regulate gene expression and are attractive candidates for biomarkers as they have been shown to play a vital role in tumorigenesis, and can be detected in clinical samples (biopsy, urine, and serum) allowing for non-invasive or minimally invasive molecular detection and prognosis of tumors [16]. Further, due to their small size they are stable in formalin-fixed paraffin-embedded (FFPE) tissues, which allows for discovery retrospectively in patient specimens [17]. Recently, numerous studies have been published demonstrating the value of studying miRNAs in the context of PCa (see ref [18] for an extensive review). These studies have used global profiling to examine miRNA signatures that are diagnostic or prognostic of biochemical failure post-prostatectomy [19–24]. However, these analyses have not accounted for treatment effects within the patient cohort. In the current study, we sought to examine a cohort of prostatectomy FFPE specimens from patients who all received salvage RT following biochemical failure post-prostatectomy using NanoString technology [25]. To our knowledge, no studies have been reported using the NanoString nCounter system to measure miRNA expression in prostate tumor specimens, which directly measures 800 human transcripts by digital counts [25]. In addition, NanoString has shown to be a better technique than qRT-PCR for FFPE specimens and correlates more with its paired fresh-frozen counterpart than qRT-PCR [26]. NanoString technology also has been used in biofluids. For example, one previous study has examined a 20-gene mRNA panel using the NanoString system for urine samples collected from patients with PCa as a potential diagnostic tool [27]. In addition, a biomarker signature utilizing NanoString technology is FDA approved and in current clinical use for breast cancer representing its clinical utility [28]. Herein, we report on miRNAs that identify PCa patients at high risk of biochemical relapse after RP and miRNAs associated with high risk of biochemical relapse in patients undergoing salvage radiation after documented biochemical failure following prostatectomy. We also present a miRNA- and clinical-based predictive model of biochemical recurrence risk post-salvage radiation therapy. To our knowledge, this article represents the first such report on the prognostic value of miRNAs in the setting of biochemical failure and salvage RT post-RP.

## Materials and Methods

### Patient Cohort

FFPE blocks from 43 patients with PCa, who had undergone RP (1997–2009) followed by salvage RT between 2005 and 2011 at the University of Freiburg, Germany, were used in this study. Patients who received adjuvant RT (RT initiated less than 6 months after RP) were excluded from the analysis. Follow-up time after RT was defined to be greater than 4 years. A clinical database was established, which contained patient characteristics, tumor classification according to the International Union Against Cancer (UICC)/American Joint Committee on Cancer (AJCC) 2010 TNM system [29,30] due to the dates of the earliest radical prostatectomies in the study, tumor grading according to Gleason score without inclusion of tertiary score, radiation treatment details including toxicities, and follow-up details including PSA concentrations. There were no significant differences in terms of radiation techniques, planning target volume generation, doses and radiation volumes in the cohort. All patients were treated 1.8–2.0 Gy per fraction to a total dose of 66.6–74 Gy. Use of hormone therapy and whole pelvis radiation was at the discretion of the treating physician. Recurrence was defined as either biochemical failure (BF) with a rise of PSA ≥ 0.2 ng/ml at least twice as per the AUA recommendation [31] or clinical progression with local (prostatic fossa), regional (lymph nodes) or distant (metastasis) recurrence. Imaging tests (CT, PET/CT or bone scan) were performed when clinically indicated in cases of BF.

**Ethics Statement.** This study was approved by both the Medical Center University Freiburg and The Ohio State University institutional review boards with waived patient consent due to the archival nature of the study. In addition, data and tissue samples pertaining to this study were de-identified prior to analysis.

### Sample Processing & RNA Isolation

All formalin-fixed, paraffin-embedded (FFPE) blocks were reviewed by the same pathologist specialized in genitourinary diseases and Gleason scores were reassessed for each core using the ISUP 2005 grading system [32]. Tissue samples from the Medical Center University of Freiburg pathology tissue bank were obtained, which included tissues from prostate tumors. Areas of interest were marked on an H&E stained slide and 1-mm diameter biopsy cores were punched from each area of interest. Cores from tumor were taken from the area with the highest Gleason Score and were enriched for tumor cells (>70%).

Total RNA was isolated using a combination of RecoverAll Total Nucleic Acid Isolation digestion buffer, AM1975, (Life Technologies; Carlsbad, CA) with Qiagen FFPE miRNeasy kit, Cat. # 217504 (Qiagen; Venlo, Limburg). In brief, cores were digested overnight using RecoverAll Total Nucleic Acid Isolation digestion buffer and proteinase K leading to enhanced digestion of the core. Once cores were digested (next day), we followed the Qiagen FFPE miRNeasy manual starting at the 80°C for 15 minute incubation. RNA concentrations were measured using a NanoDrop 2000 spectrophotometer (Thermo Scientific; Waltham, MA).

### miRNA Expression Analysis

For miRNA expression data generation, the NanoString human v2 array, which contains 800 miRNA probes, was used. Forty three tumor samples were analyzed. Expression analysis was conducted at The Ohio State University Nucleic Acid Core Facility (Columbus, OH). A total of 100ng RNA input was used per sample and conditions were set according to the manufacturer’s recommended protocol (NanoString Technologies; Seattle, WA). miRNAs were quantified using the nCounter Digital Analyzer as counts. miRNAs were filtered out from downstream analysis if total counts were less than 32 across 90% of the samples. Four hundred and seventy seven miRs were left after the filtering. Data were normalized by the geometric mean of all targets using the nSolver software (NanoString Technologies; Seattle, WA) [33]. The data discussed in this publication have been deposited in NCBI's Gene Expression Omnibus [34] and are accessible through GEO Series accession number GSE65061.

### Statistical Analysis

To compare the difference in tumor miRNA expression between late and early time to first biochemical recurrence post-RP (36 months as a cut-off) or between the time to the recurrence post-salvage RT 2-sample t-tests were used. For each miRNA, patients were dichotomized into high and low groups based on the median miRNA expression and the difference in the probabilities of the time to the recurrence (post-RP or post-salvage RT recurrence) was compared using the log-rank test for each miRNA. Multivariate analyses were performed using Cox proportional hazard regression models. ROC curve analysis was performed to determine the capability and cut-off level of variables that distinguished between the recurrence and non-recurrence of post-salvage RT. All analyses were performed using SAS 9.3 (SAS, Inc; Cary, NC) or R 3.0.

## Results

### miRNAs predictive of PSA recurrence post-prostatectomy

**Fig 1:**
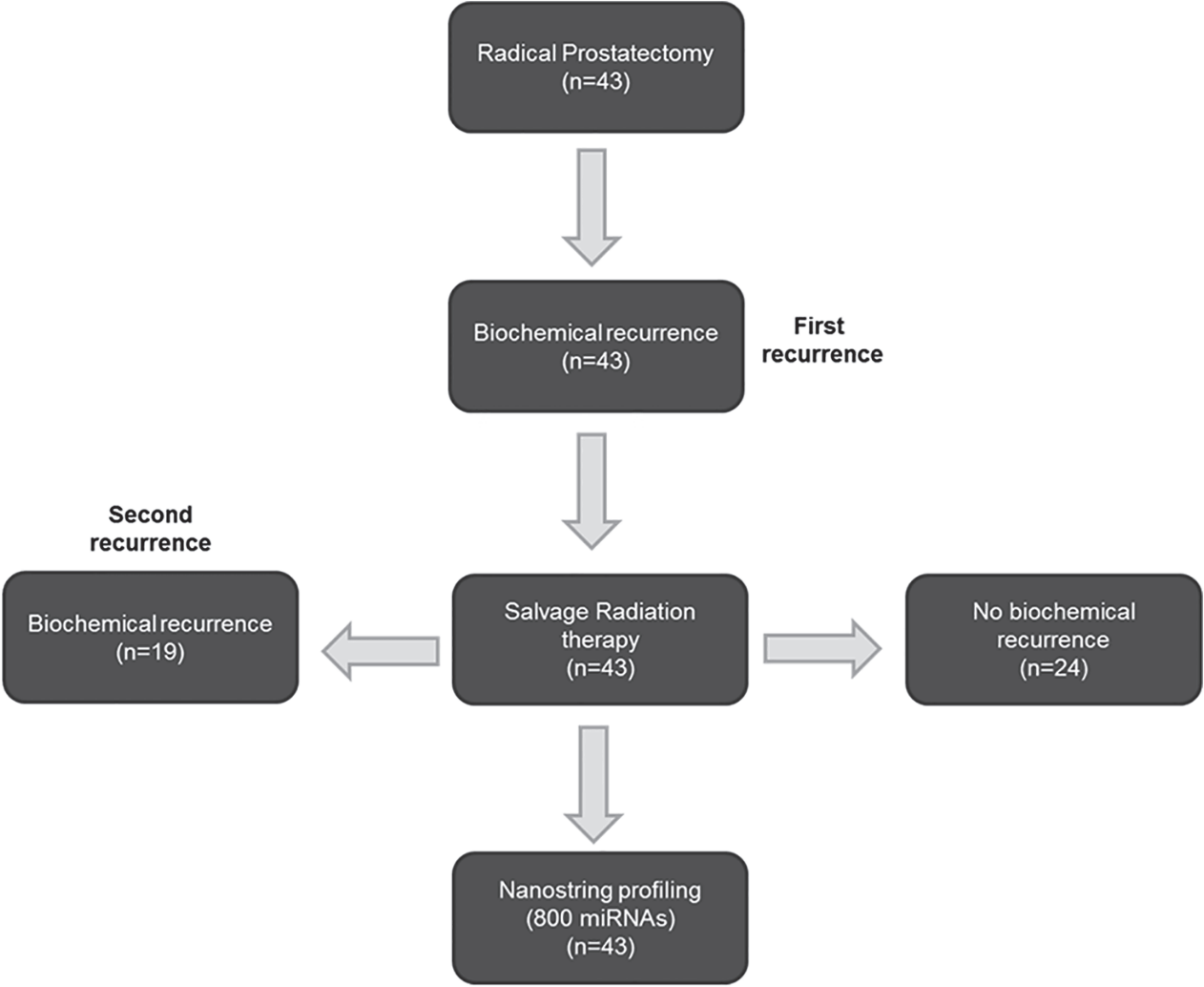
Study design. All patients underwent radical prostatectomy and salvage radiation therapy following biochemical recurrence. Tissue isolated at the time of radical prostatectomy (n = 43) was used for miRNA profiling.

**Univariate Analysis.** A schematic of the study design is shown in Fig. 1. A clinical database was established for 43 PCa patients who underwent both RP and salvage RT (Table 1). Risk factors included 32.5% of patients who had a Gleason score of 8 or above, 25.6% of patients who had seminal vesicle invasion or extraprostatic extension, and 41.9% of patients who had positive margins. Salvage RT was started at a median PSA value of 0.39 ng/ml. Nineteen patients (44.2%) experienced BF after salvage RT at a median time of 27.1 months (range 0.0–64.1 months). The median follow-up times after RP and after salvage RT were 6.9 and 3.7 years, respectively.

**Table 1:** Clinical characteristics of 43 prostate cancer patients treated with salvage radiation therapy post-prostatectomy.

	*PCa patients (n = 43)*
*Age at RP (years)* Median (min-max)	65 (45–73)
*Follow-up post-RP (years)* Median (min-max)	6.9 (4.0–13.9)
*Gleason score*	
6	8 (18.6%)
7	21 (48.8%)
8	8 (18.6%)
9	5 (11.6%)
10	1 (2.3%)
*Pathological Tumor Stage*	
T2	32 (74.4%)
T3	11 (25.6%)
*Pathological N Stage*	
N0	39 (89.0%)
N+	4 (11.0%)
*Resection Status*	
*R0*	20 (46.5%)
*R1*	18 (41.9%)
*Rx*	5 (11.6%)
*PSA at initial diagnosis (ng/mL)*< 10	30 (69.8%)
10–20	10 (23.2%)
> 20	3 (7.0%)
*Risk groups (D’Amico)*	
Low	0 (0.0%)
Intermediate	8 (18.6%)
High	35 (81.4%)
*Risk groups (Stephensen)*	
Low	25 (58.1%)
Intermediate	7 (16.3%)
High	7 (16.3%)
High +	4 (9.3%)
*Androgen Deprivation Therapy*	
Pre-op	6 (13.9%)
Pre-RT	3 (7.0%)
Concurrent	4 (9.3%)
*Time from RP to RT (months)* Median (min-max)	34.5 (10.4–123.1)
*Follow-up post-RT (years)* Median (min-max)	3.7 (0.7–7.4)
*Pre-RT PSA (ng/mL)*	
< 0.2	6 (14.0%)
0.2–1.0	28 (65.1%)
1.0–5.0	7 (16.2%)
> 5.0	2 (4.7%)
*Time from RT to BF (months)* Median (min-max)	27.1 (0.0–64.1)
*Recurrence following RT*	
Biochemical	19 (44.2%)
Proven by imaging	7 (16.3%)
Inside RT field	0 (0.0%)
Outside RT field	7 (16.3%)

RP, radical prostatectomy; min, minimum; max, maximum; RT, radiation therapy; op, operation; PSA, prostate-specific antigen.

Using these data, miRNA expression was correlated with time to first biochemical recurrence to determine if miRNAs can predict biochemical recurrence post-RP. Time to first recurrence was defined as the time from prostatectomy to the start date of salvage RT. Using tumor expression only, we identified 54 miRNAs that were significantly differentially expressed 1.5-fold or greater between patients who had an early (≤ 36 months) versus late (> 36 months) recurrence (S1 Table). Thirty six months was used as the cut-off as it was near the median of time to recurrence. For another approach to identify miRNAs that correlated with time to first biochemical recurrence, we dichotomized patients according to median miRNA expression and compared the probabilities in the time to the first recurrences between the two groups (high vs low miRNA expression) using log-rank tests. One hundred and twenty three miRNAs were identified that could differentiate the two groups in the time to first biochemical recurrence (p-value < 0.05) (S2 Table).

**Fig 2:**
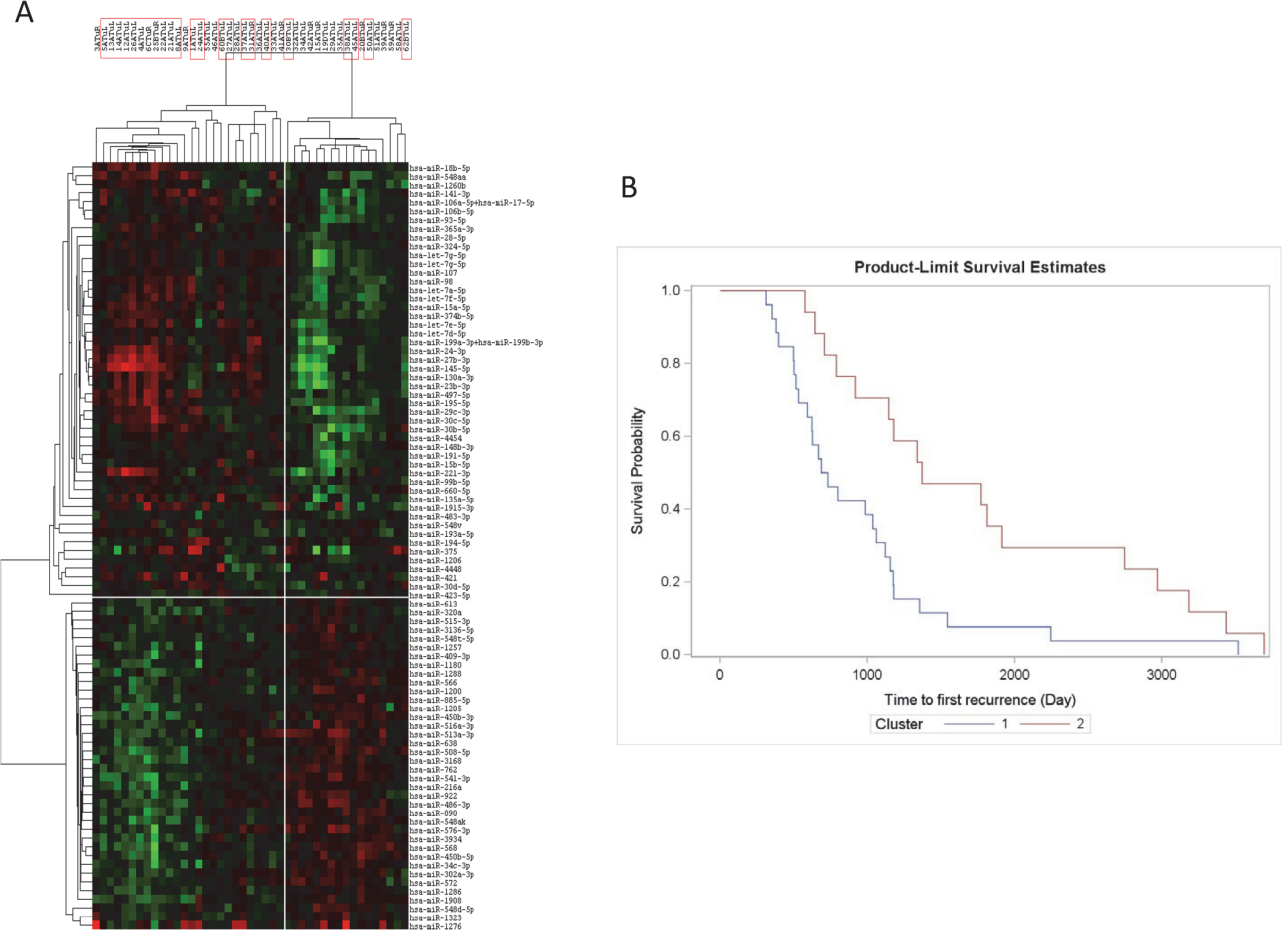
88-miRNA signature predicts early vs late biochemical recurrence post-RP. Cluster analysis was performed using the statistically significant 88 miRNAs predictive of first biochemical recurrence using Cox regression multivariable analysis (D’Amico score, Stephensen score (categorical), and Stephensen score (continuous)). This signature (A) appears to differentiate between patients with early recurrence (< 36 months) (red box) vs those with late recurrence (> 36 months). Kaplan-Meier plots were generated using the two cluster groups (B). Cluster 1 (blue), left cluster/early recurrence group; Cluster 2 (red), right cluster/late recurrence group.

**Multivariate Analysis.** In order to determine if miRNAs can be independent markers of biochemical recurrence post-RP, two multivariate Cox regression analyses were performed using all 123 miRNAs identified to be predictive of biochemical failure post-prostatectomy (p-value < 0.05) by univariate log-rank analysis (S2 Table). The first multivariate analysis considered initial PSA value (continuous) and Gleason score; age and resection status were eliminated as these did not seem to affect the recurrence in univariate analysis. Upon analysis of the first recurrence using multivariable Cox regression analysis, 97 miRNAs had p-values < 0.05 (S3 Table). In the second multivariate analysis, different clinical factors were taken into consideration. Three clinical covariates were examined independently, D’Amico score, Stephenson score (categorical), and Stephenson score (continuous), to identify miRNAs associated with time to first recurrence. miRNAs were removed from further analysis if a significant p-value (< 0.05) was not obtained in any of the three analyses which resulted in 88 miRNAs that were statistically associated with time to first recurrence after multivariate analysis (S4 Table). Patients were classified into two groups using unbiased hierarchical cluster analysis for those selected 88 miRNAs (Fig. 2A). The Kaplan-Meier method showed that the probabilities in the time to first biochemical failure were significantly different between the two groups of patients with distinguished miRNA signatures (log-rank, p = 0.005) (Fig. 2B).

### miRNAs predictive of PSA recurrence post-salvage RT

**Univariate Analysis.** miRNAs that could predict biochemical failure after salvage RT treatment are of utmost interest. At this time, no studies have examined miRNAs associated with clinical outcomes following post-RP salvage radiation. The recurrence after salvage radiation treatment (also referred to as the 2^nd^ recurrence) was defined as a rise in PSA to ≥ 0.2 ng/mL at least twice consecutively following the nadir [35]. In our patient cohort, 19 out of 43 patients experienced biochemical failure after salvage radiotherapy (Table 1). Further, using tumor miRNA expression only, we identified 4 miRNAs that were significantly expressed 1.5-fold or greater in patients who experienced a second recurrence when compared with those who did not (p-value < 0.05) (S5 Table). In the univariate log-rank analysis, 24 miRNAs were identified, which could predict patients who recurred a second time after salvage RT from those who did not recur (p-value < 0.05) (S6 Table).

**Multivariate Analysis.** Similarly, in order to determine if miRNAs can independently predict biochemical recurrence post-salvage RT, multivariate Cox regression analyses were performed using the 24 miRNAs that were significantly associated (p-value < 0.05) with recurrence post-salvage RT by univariate log-rank analysis (S6 Table). Due to the small sample size and number of events, only two covariates were examined in the multivariate analysis. The multivariate analysis was performed using lymph node status and Gleason score (these factors were chosen as having the lowest p-values upon univariate analysis as shown in (Table 2)), leading to identification of 9 miRNAs that predicted biochemical recurrence post-salvage RT (Table 3).

**Table 2:** Univariate analysis for molecular and clinical variables.

	Time to Failure Post-RP	Failure Post-Salvage RT
	p-value	p-value
88 miRNA cluster	0.005	n/a
Predictive Salvage RT Model	n/a	<0.001
Gleason Score	0.36	0.02
Lymph Node Involvement	0.11	0.001
Positive Resection Status	0.99	0.13
Pathologic Tumor Stage	0.10	0.38
PSA	0.63 (pre-op)	0.29(pre-RT)
D’Amico	0.01	0.88
Stephenson (post-RP)	0.002	0.04

Univariate log-rank tests were performed using the newly identified 88-miRNA cluster of failure post-RP and the newly developed predictive salvage radiation therapy (RT) model as well as several clinical factors to determine the ability of each factor to predict time to failure post-radical prostatectomy (RP) and failure post-salvage RT. Prostate specific antigen (PSA) and age were treated as continuous variables whereas other clinical factors were categorized as follows: Gleason score: 6, 7, and ≥8; lymph node: positive or negative; positive surgical margins: R0, R1 and Rx; pathological T stage: (T2, T2a, and T2b), (T2c), and (T3a and T3b); D’Amico risk classification: high and intermediate; Stephensen risk classification: high plus, high, intermediate, and low. n/a, not applicable.

**Table 3:** miRNAs that correlate with biochemical recurrence after salvage radiation in PCa patients.

miR-ID	Hazard Ratio (High vs. Low)	p-value	Confidence Interval	Known vs novel in PCa	Role (s) in other cancers
hsa-miR-628-3p	6.6	0.0036	1.9–23.5	Known, -5p is downregulated in serum of PCa patients [36]	Yes, [37,38]
**hsa-miR-1193**	**5.0**	**0.0064**	**1.6–15.6**	**Novel**	**No**
hsa-miR-601	4.6	0.0037	1.6–12.7	Known, contained in PC-3 exosomes [39].	Yes, [40–44]
**hsa-miR-4516**	**3.6**	**0.0128**	**1.3–10**	**Novel**	**Yes, [45]**
hsa-miR-320e	3.2	0.0339	1.1–9.6	Known, suppresses stem cell-like characteristics via Wnt/β-catenin [46]; pro-angiogenic in zebrafish/tumor xenograft model[47] upregulated in prostate tumors [48].	Yes, [49–61]
hsa-miR-508-3p	3.0	0.0296	1.1–8	Known, -5p downregulated in bone metastases vs primary PCa [62]	Yes, [63–68]
hsa-miR-598	0.3	0.0304	0.1–0.9	Known, decreased in cells and exosomes of docetaxel-resistant PCa cell lines [69]	Yes, [40,70–73]
**hsa-miR-626**	**0.3**	**0.0391**	**0.1–0.9**	**Novel**	**Yes, [69,74]**
hsa-miR-563	0.3	0.0228	0.1–0.8	Known, downregulated in urine of PCa patients [75]	Yes, [49,76,77]

Hazards ratios were generated using a multivariate Cox regression analysis (lymph node status and Gleason score). Literature supporting their role(s) in prostate cancer (PCa) as well as other cancers are referenced. Bolded are those miRNAs that are novel to PCa. Only miRNAs with a significant p-value (<0.05) are shown. CI, confidence interval

### Predictive effect of miR-601 + miR-4516 & Development of a miRNA-based Predictive Salvage RT Model

**Fig 3:**
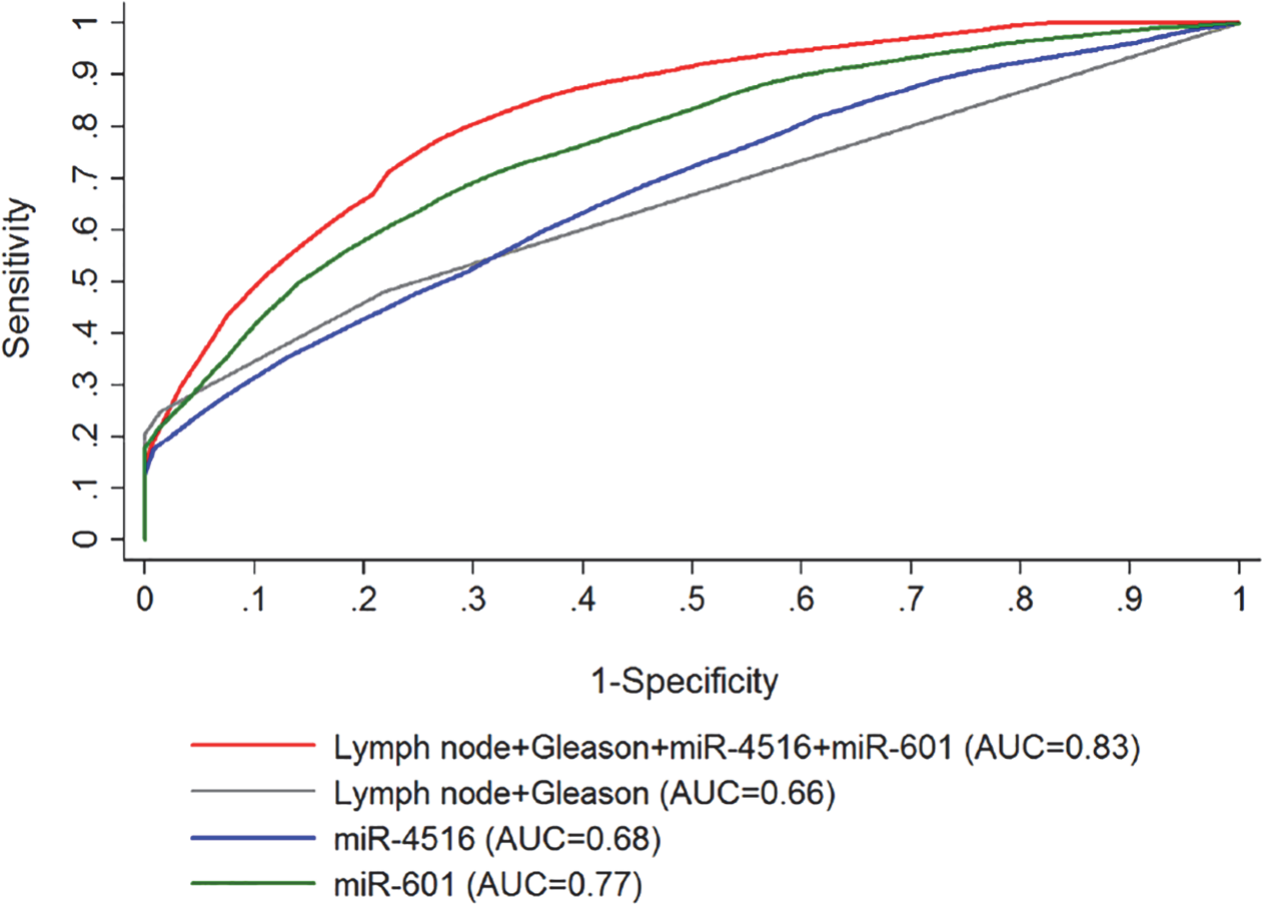
Area under the receiver operating characteristic curve (AUC) of miRNA-based predictive salvage RT model. AUC curves were generated using a stepwise Cox regression model to determine a signature predictive of biochemical recurrence post-salvage radiation therapy. Gleason score, lymph node status, hsa-miR-4516, and hsa-miR-601 (red) performs the best with an AUC of 0.83 followed by a model containing hsa-miR-601-alone (green) (AUC = 0.77), hsa-miR-4516-alone (blue) (AUC = 0.68), and lastly, Gleason score and lymph node status (grey) (AUC = 0.66).

**Fig 4:**
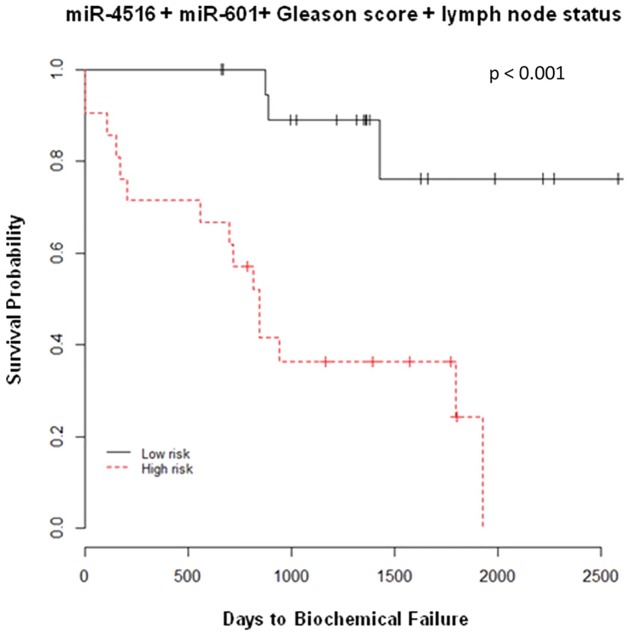
Kaplan-Meier plot estimates of miRNA-based predictive salvage RT model. A K-M plot was generated using the miR-4516 + miR-601 + Gleason score + lymph node status model for biochemical recurrence post salvage radiation therapy. Patients were divided into high and low risk groups dichotomized by the median risk score.

A primary objective of this study was to identify miRNAs that could be used independently as predictive biomarkers of salvage RT as well as to utilize in combination with other clinical factors to improve the sensitivity and selectivity of existing models. We then included lymph node status, Gleason score and the 9 miRNAs as the covariates in the Cox regression model and used a stepwise model selection strategy to capture miRNAs which could be good candidates in predicting the biochemical recurrence post-salvage RT. The two miRNAs, miR-601 and miR-4516 alone and together with Gleason score and lymph node status were selected by the model. An area under the curve (AUC) of receiver-operator characteristics (ROC) models using the nearest neighbor estimation method was utilized [78]. An AUC for lymph node and Gleason score was found to be 0.66, the addition of miR-601 and miR-4516 increased the AUC to 0.83 (Fig. 3). Interestingly, each miRNA alone (miR-601 AUC = 0.77 and miR-4516 AUC = 0.68) or together (miR-601 + miR-4516 AUC = 0.76) (Fig. 3) had a better predictive capability than the combination of positive lymph nodes together with Gleason score (AUC = 0.66) (Fig. 3). Using the risk score generated by this model (miR-4516, miR-601, Gleason score, and lymph node status), patients were classified as high or low risk groups based on median, the probabilities in recurrence post-salvage RT of the two groups were found to be significantly different (log-rank, p<0.001) as shown in Fig. 4. To ensure that hormonal therapy did not confound the predictive model, patients that received hormonal treatment at any point were removed from the model and the model still retained its specificity and sensitivity with a log-rank p-value of 0.016 and AUC value of 0.83 (S1 Fig.). To better understand mechanism(s) by which these miRNAs are acting, mRNA targets of all novel miRNAs, including miR-601 and miR-4516 were identified using TargetScan [79] and microRNA.org [80] (S7–S10 Tables).

## Discussion

Currently used models and nomograms of clinical response following RP are not able to clearly distinguish subgroups of patients with a more aggressive type of PCa from those with indolent disease. Further, there remains an ongoing debate of which patients should receive adjuvant versus salvage RT post-RP. Thus, the identification of new biomarkers that are predictive of failure post-RP is essential as PSA can only accurately identify biochemical recurrence once failure has occurred. This study sought to use miRNAs as molecular biomarkers in conjunction with clinical factors to help identify patients who would respond to salvage RT as well as patients who may be excellent candidates for adjuvant RT.

miRNAs are ideal biomarkers and capable of being detected in biopsies and biofluids which may make identification of miRNAs in PCa a clinically useful test. Very few studies have examined miRNAs globally in PCa, specifically in correlation to clinical outcome. The current study is the first to use Nanostring to profile miRNAs in PCa tissue and further elucidates the differential expression of miRNAs in PCa and correlates the expression with outcome data. There are a number of studies that have dealt with this question resulting in inconsistent results so far. One of the major problems in conducting global miRNA expression correlative studies is that a lot of the patient cohorts are heterogeneous in terms of treatments received (surgery, radiation, hormonal therapy). To our knowledge, there are no studies that have previously examined miRNAs in the context of post-prostatectomy radiation. This study addressed three different questions associated with PCa and miRNAs: 1) Which miRNA signatures can predict time to recurrence after RP?; 2) Which miRNAs can predict recurrence after salvage RT?; and 3) Which miRNAs can add predictive information beyond currently used clinico-histopathological factors?

An 88-miRNA signature was identified, which could distinguish early from late biochemical failure patients (Fig. 2). Several other studies have also investigated the prognostic ability of miRNAs in PCa and have shown that alone or in combination with other clinical factors, miRNAs serve as good predictive biomarkers of clinical outcome [19–24,81–83]. Of the 88 miRNAs in our signatures, 15 were previously reported to be a part of a PCa prognostic signature or model. These include miR-145-5p [20,21], miR-141-3p [21,22], miR-27b-3p [21,81], miR-106b-5p, miR-93-5p [21] miR-148a-3p, miR-193a-3p [22,81], miR-135a-5p, miR-374b-5p, miR-29c-3p, miR-365a-3p [22], let-7a-5p, miR-515-3p [24], and miR-34c-3p [81]. Pathway analysis was performed using Ingenuity Pathway Analysis (IPA) for these 88-miRNAs and the top network identified included cancer, gastrointestinal disease, and respiratory disease. The 88-miRNA signature was also predicted to be involved in important molecular and cellular functions pertaining to cancer (S11 Table).

As previously indicated, the identification of miRNAs associated with outcome after salvage RT is the most novel and impactful part of this study. Of the 9 miRNAs associated with biochemical recurrence after salvage RT (Table 3), all except miR-1193 have been reported to be associated with cancer in general [37,38,40–45,49–61,63–74,76,77,84]. Six miRNAs (miR-601, miR-628-3p, miR-320e, miR-508-3p, miR-598, and miR-563) have been previously linked to PCa [36,39,46–48,62,75,85] and of those, only miR-320e has shown functional relevance in *in vitro* studies [46,47]. Thus, we have identified 3 novel PCa miRNAs (miR-1193, miR-4516, and miR-626) associated with second biochemical recurrence after salvage RT. Because these miRNAs are novel to PCa, we wanted to investigate the putative mRNA targets of miR-1193, miR-4516, and miR-626. TargetScan and microRNA.org were used to determine the mRNA targets for each of the miRNAs (S7–S10 Tables). Pathway analysis was performed using IPA for all 9-miRNAs associated with second biochemical recurrence and the top network identified included cancer, cell morphology, and cell assembly and organization (S12 Table).

This study identified the first miRNA-based predictive model for biochemical recurrence following salvage RT. The two miRNAs that were significant within the predictive model were miR-601 and miR-4516; both of which were upregulated in patients who experienced biochemical failure post-salvage RT. Currently, there are no publications regarding miR-4516 and PCa. In March of 2014, the only cancer-related study for miR-4516 was published in which Chowdhari et al. showed that miR-4516 downregulates STAT3 and mediates UV-induced apoptosis in a human keratinocyte cell line [45]. As for miR-601, it has been shown to be overexpressed in both gastric [42] and esophageal squamous cell carcinoma tumor tissues [43]. miR-601 has been shown to be a diagnostic serum biomarker for colorectal cancer [44] and has been found to target and downregulate B7-H3, a immunoregulatory protein associated with poor prognosis and metastasis in several cancers and in breast cancer cell lines [41]. Importantly, for this study, miR-601 has been shown to be differentially expressed post-radiation in human lymphocytes [40] and has been identified in PC-3 released exosomes *in vitro* [39]. A potential mechanism for the role of miR-601 and radiation outcomes maybe the involvement of miR-601 in the regulation of the NF-kB pathway [86], an inflammatory signaling pathway that has been shown by multiple groups to be important for radiation response [87,88]. Additional putative targets of both miR-601 and miR-4516 are shown in S8–S10 Tables and proteins in multiple pathways including apoptosis and cell cycle regulation were identified. Although miR-601 appears to be a major player in predictive ability of salvage radiation response, miR-4516 and miR-601 together appear to be correlated (Pearson r = 0.56, p-value <0.0001) and target pathways of both STAT3 and NF-kB pathways that are critical regulators of the inflammatory responses in cancer [89]. This study warrants further functional characterization of these miRNAs in correlation to radiation response in PCa. These future analyses will help elucidate the roles that both miR-4516 and miR-601 have in the clinical response to salvage-RT post-prostatectomy.

There are some limitations to the present study. First of all the sample size was relatively small, secondly, <50% of patients had BF after salvage RT, and lastly, androgen deprivation therapy was prescribed at the discretion of the treating physician for a small subset of patients reflecting current treatment practices but imposing bias to the study cohort. Although hormonal therapy does not appear to be a confounding variable in the study, further validation using larger samples sizes as well as patients who received adjuvant and observation after prostatectomy will be required to determine the clinical usefulness of the identified predictive models. However, the predictive ability of the identified signature and regression model appears to be potentially useful relative to currently used clinical and histo-pathological criteria. In addition, further investigation of molecular mechanisms involving miRNAs will likely lead to the elucidation of prostate tumorigeneisis and progression as well as identification of putative targets for therapy.

In summary, a miRNA signature was identified that may be able to predict time to biochemical recurrence following RP and a logistic regression model was established which predicts biochemical failure following salvage RT. The 88-miRNA signature alone has the potential to predict time to first recurrence indicating that miRNAs identified by NanoString using FFPE specimens can serve as promising biomarkers of biochemical failure prediction. Currently, there are no routinely used clinical factors or models to predict failure post-salvage RT. We showed that miRNAs alone predicted biochemical recurrence post-salvage RT and adding two miRNAs, miR-601 and miR-4516, to lymph node status and Gleason score, greatly improved the predictive ability of these clinical factors alone. Additionally, this post-RT predictive model was superior to other clinical factors examined in this cohort of patients.

## Supporting Information

S1 Fig Kaplan-Meier plot estimates and AUC values of miRNA-based predictive salvage RT model without patients treated with hormonal therapy. Patients were divided into high and low risk groups dichotomized by the median risk score.
(DOCX)

S1 Table Tumor-only miRNA expression comparisons between patients with early (≤36 months) and late (>36 months) biochemical recurrence. p-values were calculated using a repeated measured ANOVA model.
(DOCX)

S2 Table Tumor-only miRNA expression was used to predict time to first biochemical recurrence. Patients were divided into two groups by the median miRNA expression and a univariate log-rank test was performed to correlate expression with time to first biochemical recurrence. Significant p-value < 0.05.
(DOCX)

S3 Table miRNAs that predict time to failure post-RP (first recurrence) in salvage radiation patients. Hazards ratios were generated using a multivariate Cox regression analysis (initial PSA and Gleason score). Only miRNAs with a significant p-value (<0.05) are shown.
(DOCX)

S4 Table miRNAs that predict time to failure post-RP (first recurrence) in salvage radiation patients. Hazard ratios were generated using a multivariate Cox regression analysis (D’Amico, categorical Stephensen, or continuous Stephensen). Only miRNAs with a significant p-value (<0.05) are shown.
(DOCX)

S5 Table Tumor-only miRNA expression comparisons between patients with biochemical recurrence post-salvage RT (second biochemical recurrence) versus no recurrence post-salvage radiation therapy. p-values were generated using a repeated measured ANOVA.
(DOCX)

S6 Table Tumor-only miRNA expression was used to predict biochemical recurrence post-salvage RT (second biochemical recurrence). Patients were divided into two groups by the median miRNA expression and a univariate log-rank test was performed to correlate expression with second biochemical recurrence. Significant p-value < 0.05.
(DOCX)

S7 Table Putative targets of novel miRNAs, miR-1193 and miR-626 associated with biochemical recurrence post-salvage RT (second biochemical recurrence). All targets of both miR-1193 and miR-626 are listed with scores according to both TargetScan and microRNA.org.
(DOCX)

S8 Table Putative targets of miRNAs, miR-601 and miR-4516, within our predictive model of biochemical recurrence post-salvage RT (second biochemical recurrence). All targets of both miR-601 and miR-4516 are listed with scores according to both TargetScan and microRNA.org. miR-4516 was not listed in microRNA.org.
(DOCX)

S9 Table Top 12 putative gene targets and function for miR-601. Twelve genes with the lowest scores and highest probability of being targeted by miR-601 according to TargetScan and microRNA.org are listed with their known gene functions.
(DOCX)

S10 Table Top 10 putative gene targets and function for miR-4516. Ten genes with the lowest scores and highest probability of being targeted by miR-4516 according to TargetScan are listed with their known gene functions.
(DOCX)

S11 Table Ingenuity Pathway Analysis (IPA) was performed on the 88-miRNA signature associated with time to first biochemical recurrence. Displayed are top networks, diseases and disorders, and molecular and cellular functions.
(DOCX)

S12 Table Ingenuity Pathway Analysis (IPA) was performed on the 9-miRNAs associated with biochemical recurrence post-salvage radiation therapy. Displayed are top networks and diseases and disorders.
(DOCX)

## References

[1] Amling CL, Blute ML, Bergstralh EJ, Seay TM, Slezak J, Zincke H. Long-term hazard of progression after radical prostatectomy for clinically localized prostate cancer: continued risk of biochemical failure after 5 years. J Urol. 2000; 164: 101–105. 10840432

[2] Amling CL. Biochemical recurrence after localized treatment. Urol Clin North Am. 2006; 33: 147–159, v. 16631453 10.1016/j.ucl.2005.12.002

[3] Botticella A, Guarneri A, Levra NG, Munoz F, Filippi AR, Rondi N, et al Biochemical and clinical outcomes after high-dose salvage radiotherapy as monotherapy for prostate cancer. J Cancer Res Clin Oncol. 2014; 140: 1111–1116. 10.1007/s00432-014-1673-8 24744191 PMC11824060

[4] Cooperberg MR, Freedland SJ, Pasta DJ, Elkin EP, Presti JC Jr, Amling CL, et al Multiinstitutional validation of the UCSF cancer of the prostate risk assessment for prediction of recurrence after radical prostatectomy. Cancer. 2006; 107: 2384–2391. 17039503 10.1002/cncr.22262

[5] May M, Knoll N, Siegsmund M, Fahlenkamp D, Vogler H, Hoschke B, et al Validity of the CAPRA score to predict biochemical recurrence-free survival after radical prostatectomy. Results from a european multicenter survey of 1,296 patients. J Urol. 2007; 178: 1957–1962; discussion 1962. 17868719 10.1016/j.juro.2007.07.043

[6] Cooperberg MR, Pasta DJ, Elkin EP, Litwin MS, Latini DM, Du Chane J, et al The University of California, San Francisco Cancer of the Prostate Risk Assessment score: a straightforward and reliable preoperative predictor of disease recurrence after radical prostatectomy. J Urol. 2005; 173: 1938–1942. 15879786 10.1097/01.ju.0000158155.33890.e7 PMC2948569

[7] Makarov DV, Trock BJ, Humphreys EB, Mangold LA, Walsh PC, Epstein JI, et al Updated nomogram to predict pathologic stage of prostate cancer given prostate-specific antigen level, clinical stage, and biopsy Gleason score (Partin tables) based on cases from 2000 to 2005. Urology. 2007; 69: 1095–1101. 17572194 10.1016/j.urology.2007.03.042 PMC1993240

[8] Partin AW, Mangold LA, Lamm DM, Walsh PC, Epstein JI, Pearson JD. Contemporary update of prostate cancer staging nomograms (Partin Tables) for the new millennium. Urology. 2001; 58: 843–848. 11744442 10.1016/s0090-4295(01)01441-8

[9] D'Amico AV, Whittington R, Malkowicz SB, Wu YH, Chen M, Art M, et al Combination of the preoperative PSA level, biopsy gleason score, percentage of positive biopsies, and MRI T-stage to predict early PSA failure in men with clinically localized prostate cancer. Urology. 2000; 55: 572–577. 10736506 10.1016/s0090-4295(99)00479-3

[10] Stephenson AJ, Scardino PT, Eastham JA, Bianco FJ Jr, Dotan ZA, DiBlasio CJ, et al Postoperative nomogram predicting the 10-year probability of prostate cancer recurrence after radical prostatectomy. J Clin Oncol. 2005; 23: 7005–7012. 16192588 10.1200/JCO.2005.01.867 PMC2231088

[11] Stephenson AJ, Scardino PT, Eastham JA, Bianco FJ Jr, Dotan ZA, Fearn PA, et al Preoperative nomogram predicting the 10-year probability of prostate cancer recurrence after radical prostatectomy. J Natl Cancer Inst. 2006; 98: 715–717. 16705126 10.1093/jnci/djj190 PMC2242430

[12] Stephenson AJ, Scardino PT, Kattan MW, Pisansky TM, Slawin KM, Klein EA, et al Predicting the outcome of salvage radiation therapy for recurrent prostate cancer after radical prostatectomy. J Clin Oncol. 2007; 25: 2035–2041. 17513807 10.1200/JCO.2006.08.9607 PMC2670394

[13] Knezevic D, Goddard AD, Natraj N, Cherbavaz DB, Clark-Langone KM, Snable J, et al Analytical validation of the Oncotype DX prostate cancer assay—a clinical RT-PCR assay optimized for prostate needle biopsies. BMC Genomics. 2013; 14: 690 10.1186/1471-2164-14-690 24103217 PMC4007703

[14] Erho N, Crisan A, Vergara IA, Mitra AP, Ghadessi M, Buerki C, et al Discovery and validation of a prostate cancer genomic classifier that predicts early metastasis following radical prostatectomy. PLoS One. 2013; 8: e66855 10.1371/journal.pone.0066855 23826159 PMC3691249

[15] Den RB, Feng FY, Showalter TN, Mishra MV, Trabulsi EJ, Lallas CD, et al Genomic prostate cancer classifier predicts biochemical failure and metastases in patients after postoperative radiation therapy. Int J Radiat Oncol Biol Phys. 2014; 89: 1038–1046. 10.1016/j.ijrobp.2014.04.052 25035207 PMC4432840

[16] Mitchell PS, Parkin RK, Kroh EM, Fritz BR, Wyman SK, Pogosova-Agadjanyan EL, et al Circulating microRNAs as stable blood-based markers for cancer detection. Proc Natl Acad Sci U S A. 2008; 105: 10513–10518. 10.1073/pnas.0804549105 18663219 PMC2492472

[17] Liu A, Xu X. MicroRNA isolation from formalin-fixed, paraffin-embedded tissues. Methods Mol Biol. 2011; 724: 259–267. 10.1007/978-1-61779-055-3_16 21370018 PMC4527651

[18] Kim WT, Kim WJ. MicroRNAs in prostate cancer. Prostate Int. 2013; 1: 3–9. 10.12954/PI.12011 24223395 PMC3821523

[19] Fendler A, Jung M, Stephan C, Honey RJ, Stewart RJ, Pace KT, et al miRNAs can predict prostate cancer biochemical relapse and are involved in tumor progression. Int J Oncol. 2011; 39: 1183–1192. 10.3892/ijo.2011.1128 21769427

[20] Larne O, Martens-Uzunova E, Hagman Z, Edsjo A, Lippolis G, den Berg MS, et al miQ—a novel microRNA based diagnostic and prognostic tool for prostate cancer. Int J Cancer. 2013; 132: 2867–2875. 10.1002/ijc.27973 23184647

[21] Martens-Uzunova ES, Jalava SE, Dits NF, van Leenders GJ, Moller S, Trapman J, et al Diagnostic and prognostic signatures from the small non-coding RNA transcriptome in prostate cancer. Oncogene. 2012; 31: 978–991. 10.1038/onc.2011.304 21765474

[22] Lichner Z, Fendler A, Saleh C, Nasser AN, Boles D, Al-Haddad S, et al MicroRNA signature helps distinguish early from late biochemical failure in prostate cancer. Clin Chem. 2013; 59: 1595–1603. 10.1373/clinchem.2013.205450 23958847

[23] Long Q, Johnson BA, Osunkoya AO, Lai YH, Zhou W, Abramovitz M, et al Protein-coding and microRNA biomarkers of recurrence of prostate cancer following radical prostatectomy. Am J Pathol. 2011; 179: 46–54. 10.1016/j.ajpath.2011.03.008 21703393 PMC3123866

[24] Schubert M, Spahn M, Kneitz S, Scholz CJ, Joniau S, Stroebel P, et al Distinct microRNA expression profile in prostate cancer patients with early clinical failure and the impact of let-7 as prognostic marker in high-risk prostate cancer. PLoS One. 2013; 8: e65064 10.1371/journal.pone.0065064 23798998 PMC3683014

[25] Geiss GK, Bumgarner RE, Birditt B, Dahl T, Dowidar N, Dunaway DL, et al Direct multiplexed measurement of gene expression with color-coded probe pairs. Nat Biotechnol. 2008; 26: 317–325. 10.1038/nbt1385 18278033

[26] Reis PP, Waldron L, Goswami RS, Xu W, Xuan Y, Perez-Ordonez B, et al mRNA transcript quantification in archival samples using multiplexed, color-coded probes. BMC Biotechnol. 2011; 11: 46 10.1186/1472-6750-11-46 21549012 PMC3103428

[27] Quek SI, Ho ME, Loprieno MA, Ellis WJ, Elliott N, Liu AY. A multiplex assay to measure RNA transcripts of prostate cancer in urine. PLoS One. 2012; 7: e45656 10.1371/journal.pone.0045656 23029164 PMC3447789

[28] Nielsen T, Wallden B, Schaper C, Ferree S, Liu S, Gao D, et al Analytical validation of the PAM50-based Prosigna Breast Cancer Prognostic Gene Signature Assay and nCounter Analysis System using formalin-fixed paraffin-embedded breast tumor specimens. BMC Cancer. 2014; 14: 177 10.1186/1471-2407-14-177 24625003 PMC4008304

[29] Greene FL, Compton CC, Fritz AG, Shah JP, Winchester DP. AJCC Cancer Staging Manual Springer 2009.

[30] Sobin LH, Gospodarowicz MK, and Wittekind C. TNM Classification of Malignant Tumours: John Wiley & Sons 2009.

[31] Thompson I, Thrasher JB, Aus G, Burnett AL, Canby-Hagino ED, Cookson MS, et al Guideline for the management of clinically localized prostate cancer: 2007 update. J Urol. 2007; 177: 2106–2131. 17509297 10.1016/j.juro.2007.03.003

[32] Epstein JI, Allsbrook WC Jr, Amin MB, Egevad LL. The 2005 International Society of Urological Pathology (ISUP) Consensus Conference on Gleason Grading of Prostatic Carcinoma. Am J Surg Pathol. 2005; 29: 1228–1242. 16096414 10.1097/01.pas.0000173646.99337.b1

[33] Mestdagh P, Van Vlierberghe P, De Weer A, Muth D, Westermann F, Speleman F, et al A novel and universal method for microRNA RT-qPCR data normalization. Genome Biol. 2009; 10: R64 10.1186/gb-2009-10-6-r64 19531210 PMC2718498

[34] Edgar R, Domrachev M, Lash AE. Gene Expression Omnibus: NCBI gene expression and hybridization array data repository. Nucleic Acids Res. 2002; 30: 207–210. 11752295 10.1093/nar/30.1.207 PMC99122

[35] Cookson MS, Aus G, Burnett AL, Canby-Hagino ED, D'Amico AV, Dmochowski RR, et al Variation in the definition of biochemical recurrence in patients treated for localized prostate cancer: the American Urological Association Prostate Guidelines for Localized Prostate Cancer Update Panel report and recommendations for a standard in the reporting of surgical outcomes. J Urol. 2007; 177: 540–545. 17222629 10.1016/j.juro.2006.10.097

[36] Srivastava A, Goldberger H, Dimtchev A, Marian C, Soldin O, Li X, et al Circulatory miR-628-5p is downregulated in prostate cancer patients. Tumour Biol. 2014; 35: 4867–4873. 10.1007/s13277-014-1638-1 24477576 PMC4663977

[37] Mazeh H, Mizrahi I, Ilyayev N, Halle D, Brucher B, Bilchik A, et al The Diagnostic and Prognostic Role of microRNA in Colorectal Cancer—a Comprehensive review. J Cancer. 2013; 4: 281–295. 10.7150/jca.5836 23459799 PMC3584841

[38] Li A, Yu J, Kim H, Wolfgang CL, Canto MI, Hruban RH, et al MicroRNA array analysis finds elevated serum miR-1290 accurately distinguishes patients with low-stage pancreatic cancer from healthy and disease controls. Clin Cancer Res. 2013; 19: 3600–3610. 10.1158/1078-0432.CCR-12-3092 23697990 PMC3707520

[39] Hessvik NP, Phuyal S, Brech A, Sandvig K, Llorente A. Profiling of microRNAs in exosomes released from PC-3 prostate cancer cells. Biochim Biophys Acta. 2012; 1819: 1154–1163. 10.1016/j.bbagrm.2012.08.016 22982408

[40] Girardi C, De Pitta C, Casara S, Sales G, Lanfranchi G, Celotti L, et al Analysis of miRNA and mRNA expression profiles highlights alterations in ionizing radiation response of human lymphocytes under modeled microgravity. PLoS One. 2012; 7: e31293 10.1371/journal.pone.0031293 22347458 PMC3276573

[41] Nygren MK, Tekle C, Ingebrigtsen VA, Makela R, Krohn M, Aure MR, et al Identifying microRNAs regulating B7-H3 in breast cancer: the clinical impact of microRNA-29c. Br J Cancer. 2014; 110: 2072–2080. 10.1038/bjc.2014.113 24577056 PMC3992492

[42] Yao Y, Suo AL, Li ZF, Liu LY, Tian T, Ni L, et al MicroRNA profiling of human gastric cancer. Mol Med Rep. 2009; 2: 963–970. 10.3892/mmr_00000199 21475928

[43] Yang M, Liu R, Sheng J, Liao J, Wang Y, Pan E, et al Differential expression profiles of microRNAs as potential biomarkers for the early diagnosis of esophageal squamous cell carcinoma. Oncol Rep. 2013; 29: 169–176. 10.3892/or.2012.2105 23124769

[44] Yang Y, Gu X, Zhou M, Xiang J, Chen Z. Serum microRNAs: A new diagnostic method for colorectal cancer. Biomed Rep. 2013; 1: 495–498. 24648974 10.3892/br.2013.109 PMC3917018

[45] Chowdhari S, Saini N. hsa-miR-4516 Mediated Downregulation of STAT3/CDK6/UBE2N Plays a Role in PUVA Induced Apoptosis in Keratinocytes. J Cell Physiol. 2014. 10.1002/jcp.24608 24610393

[46] Hsieh IS, Chang KC, Tsai YT, Ke JY, Lu PJ, Lee KH, et al MicroRNA-320 suppresses the stem cell-like characteristics of prostate cancer cells by downregulating the Wnt/beta-catenin signaling pathway. Carcinogenesis. 2013; 34: 530–538. 10.1093/carcin/bgs371 23188675

[47] Chiavacci E, Rizzo M, Pitto L, Patella F, Evangelista M, Mariani L, et al. The zebrafish/tumor xenograft angiogenesis assay as a tool for screening anti-angiogenic miRNAs. Cytotechnology. 2014. 10.1007/s10616-014-9735-y PMC4628928 24947063

[48] Gordanpour A, Nam RK, Sugar L, Seth A. MicroRNAs in prostate cancer: from biomarkers to molecularly-based therapeutics. Prostate Cancer Prostatic Dis. 2012; 15: 314–319. 10.1038/pcan.2012.3 22333688

[49] Liu X, Chen Z, Yu J, Xia J, Zhou X. MicroRNA profiling and head and neck cancer. Comp Funct Genomics. 2009: 837514. 10.1155/2009/837514 PMC2688814 19753298

[50] Schaar DG, Medina DJ, Moore DF, Strair RK, Ting Y. miR-320 targets transferrin receptor 1 (CD71) and inhibits cell proliferation. Exp Hematol. 2009; 37: 245–255. 10.1016/j.exphem.2008.10.002 19135902

[51] Lee KH, Lotterman C, Karikari C, Omura N, Feldmann G, Habbe N, et al Epigenetic silencing of MicroRNA miR-107 regulates cyclin-dependent kinase 6 expression in pancreatic cancer. Pancreatology. 2009; 9: 293–301. 10.1159/000186051 19407485 PMC2835374

[52] Yan LX, Huang XF, Shao Q, Huang MY, Deng L, Wu QL, et al MicroRNA miR-21 overexpression in human breast cancer is associated with advanced clinical stage, lymph node metastasis and patient poor prognosis. RNA. 2008; 14: 2348–2360. 10.1261/rna.1034808 18812439 PMC2578865

[53] Chen L, Yan HX, Yang W, Hu L, Yu LX, Liu Q, et al The role of microRNA expression pattern in human intrahepatic cholangiocarcinoma. J Hepatol. 2009; 50: 358–369. 10.1016/j.jhep.2008.09.015 19070389

[54] Gao W, Shen H, Liu L, Xu J, Shu Y. MiR-21 overexpression in human primary squamous cell lung carcinoma is associated with poor patient prognosis. J Cancer Res Clin Oncol. 2011; 137: 557–566. 10.1007/s00432-010-0918-4 20508945 PMC11828261

[55] Schepeler T, Reinert JT, Ostenfeld MS, Christensen LL, Silahtaroglu AN, Dyrskjot L, et al Diagnostic and prognostic microRNAs in stage II colon cancer. Cancer Res. 2008; 68: 6416–6424. 10.1158/0008-5472.CAN-07-6110 18676867

[56] Bronisz A, Godlewski J, Wallace JA, Merchant AS, Nowicki MO, Mathsyaraja H, et al Reprogramming of the tumour microenvironment by stromal PTEN-regulated miR-320. Nat Cell Biol. 2012; 14: 159–167. 10.1038/ncb2396 22179046 PMC3271169

[57] Ziliak D, Gamazon ER, Lacroix B, Kyung Im H, Wen Y, Huang RS. Genetic variation that predicts platinum sensitivity reveals the role of miR-193b* in chemotherapeutic susceptibility. Mol Cancer Ther. 2012; 11: 2054–2061. 10.1158/1535-7163.MCT-12-0221 22752226 PMC3438340

[58] Wu YY, Chen YL, Jao YC, Hsieh IS, Chang KC, Hong TM. miR-320 regulates tumor angiogenesis driven by vascular endothelial cells in oral cancer by silencing neuropilin 1. Angiogenesis. 2014; 17: 247–260. 10.1007/s10456-013-9394-1 24114198

[59] Noto JM, Piazuelo MB, Chaturvedi R, Bartel CA, Thatcher EJ, Delgado A, et al Strain-specific suppression of microRNA-320 by carcinogenic Helicobacter pylori promotes expression of the antiapoptotic protein Mcl-1. Am J Physiol Gastrointest Liver Physiol. 2013; 305: G786–796. 10.1152/ajpgi.00279.2013 24136787 PMC3882435

[60] Blenkiron C, Hurley DG, Fitzgerald S, Print CG, Lasham A. Links between the oncoprotein YB-1 and small non-coding RNAs in breast cancer. PLoS One. 2013; 8: e80171 10.1371/journal.pone.0080171 24260353 PMC3832415

[61] Cheng C, Chen ZQ, Shi XT. MicroRNA-320 inhibits osteosarcoma cells proliferation by directly targeting fatty acid synthase. Tumour Biol. 2014; 35: 4177–4183. 10.1007/s13277-013-1546-9 24390663

[62] Peng X, Guo W, Liu T, Wang X, Tu X, Xiong D, et al Identification of miRs-143 and -145 that is associated with bone metastasis of prostate cancer and involved in the regulation of EMT. PLoS One. 2011; 6: e20341 10.1371/journal.pone.0020341 21647377 PMC3103579

[63] Shang Y, Zhang Z, Liu Z, Feng B, Ren G, Li K, et al miR-508-5p regulates multidrug resistance of gastric cancer by targeting ABCB1 and ZNRD1. Oncogene. 2014; 33: 3267–3276. 10.1038/onc.2013.297 23893241

[64] Liang S, Chen L, Huang H, Zhi D. The experimental study of miRNA in pituitary adenomas. Turk Neurosurg. 2013; 23: 721–727. 10.5137/1019-5149.JTN.7425-12.1 24310454

[65] Yan H, Wang S, Yu H, Zhu J, Chen C. Molecular pathways and functional analysis of miRNA expression associated with paclitaxel-induced apoptosis in hepatocellular carcinoma cells. Pharmacology. 2013; 92: 167–174. 10.1159/000354585 24060847

[66] Wang Z, Wang J, Yang Y, Hao B, Wang R, Li Y, et al Loss of has-miR-337-3p expression is associated with lymph node metastasis of human gastric cancer. J Exp Clin Cancer Res. 2013; 32: 76 10.1186/1756-9966-32-76 24422944 PMC3854519

[67] Yu X, Zhang X, Bi T, Ding Y, Zhao J, Wang C, et al MiRNA expression signature for potentially predicting the prognosis of ovarian serous carcinoma. Tumour Biol. 2013; 34: 3501–3508. 10.1007/s13277-013-0928-3 23836287

[68] Zhai Q, Zhou L, Zhao C, Wan J, Yu Z, Guo X, et al Identification of miR-508-3p and miR-509-3p that are associated with cell invasion and migration and involved in the apoptosis of renal cell carcinoma. Biochem Biophys Res Commun. 2012; 419: 621–626. 10.1016/j.bbrc.2012.02.060 22369946

[69] Dong L, Li Y, Han C, Wang X, She L, Zhang H. miRNA microarray reveals specific expression in the peripheral blood of glioblastoma patients. Int J Oncol. 2014; 45: 746–756. 10.3892/ijo.2014.2459 24858071

[70] Mosakhani N, Guled M, Leen G, Calabuig-Farinas S, Niini T, Machado I, et al An integrated analysis of miRNA and gene copy numbers in xenografts of Ewing's sarcoma. J Exp Clin Cancer Res. 2012; 31: 24 10.1186/1756-9966-31-24 22429812 PMC3338077

[71] Skalsky RL, Cullen BR. Reduced expression of brain-enriched microRNAs in glioblastomas permits targeted regulation of a cell death gene. PLoS One. 2011; 6: e24248 10.1371/journal.pone.0024248 21912681 PMC3166303

[72] Zhao BS, Liu SG, Wang TY, Ji YH, Qi B, Tao YP, et al Screening of microRNA in patients with esophageal cancer at same tumor node metastasis stage with different prognoses. Asian Pac J Cancer Prev. 2013; 14: 139–143. 23534712 10.7314/apjcp.2013.14.1.139

[73] Yoshino H, Seki N, Itesako T, Chiyomaru T, Nakagawa M, Enokida H. Aberrant expression of microRNAs in bladder cancer. Nat Rev Urol. 2013; 10: 396–404. 10.1038/nrurol.2013.113 23712207

[74] Riaz M, van Jaarsveld MT, Hollestelle A, Prager-van der Smissen WJ, Heine AA, Boersma AW, et al miRNA expression profiling of 51 human breast cancer cell lines reveals subtype and driver mutation-specific miRNAs. Breast Cancer Res. 2013; 15: R33 10.1186/bcr3415 23601657 PMC3672661

[75] Haj-Ahmad TA, Abdalla MA, Haj-Ahmad Y. Potential Urinary miRNA Biomarker Candidates for the Accurate Detection of Prostate Cancer among Benign Prostatic Hyperplasia Patients. J Cancer. 2014; 5: 182–191. 10.7150/jca.6799 24563673 PMC3931266

[76] Cao YX, Dai CW, Zhang GS. Screening for drug resistance related microRNAs in K562 and K562/A02 cell lines. Zhonghua Xue Ye Xue Za Zhi. 2010; 31: 361–365. 21122348

[77] Volinia S, Galasso M, Costinean S, Tagliavini L, Gamberoni G, Drusco A, et al Reprogramming of miRNA networks in cancer and leukemia. Genome Res. 2010; 20: 589–599. 10.1101/gr.098046.109 20439436 PMC2860161

[78] Heagerty PJ, Lumley T, Pepe MS. Time-dependent ROC curves for censored survival data and a diagnostic marker. Biometrics. 2000; 56: 337–344. 10877287 10.1111/j.0006-341x.2000.00337.x

[79] Grimson A, Farh KK, Johnston WK, Garrett-Engele P, Lim LP, Bartel DP. MicroRNA targeting specificity in mammals: determinants beyond seed pairing. Mol Cell. 2007; 27: 91–105. 17612493 10.1016/j.molcel.2007.06.017 PMC3800283

[80] Betel D, Wilson M, Gabow A, Marks DS, Sander C. The microRNA.org resource: targets and expression. Nucleic Acids Res. 2008; 36: D149–153. 10.1093/nar/gkm995 PMC2238905 18158296

[81] Walter BA, Valera VA, Pinto PA, Merino MJ. Comprehensive microRNA Profiling of Prostate Cancer. J Cancer. 2013; 4: 350–357. 10.7150/jca.6394 23781281 PMC3677622

[82] Schaefer A, Jung M, Miller K, Lein M, Kristiansen G, Erbersdobler A, et al Suitable reference genes for relative quantification of miRNA expression in prostate cancer. Exp Mol Med. 2010; 42: 749–758. 20890088 10.3858/emm.2010.42.11.076 PMC2992854

[83] Taylor BS, Schultz N, Hieronymus H, Gopalan A, Xiao Y, Carver BS, et al Integrative genomic profiling of human prostate cancer. Cancer Cell. 2010; 18: 11–22. 10.1016/j.ccr.2010.05.026 20579941 PMC3198787

[84] Zhao JJ, Yang J, Lin J, Yao N, Zhu Y, Zheng J, et al Identification of miRNAs associated with tumorigenesis of retinoblastoma by miRNA microarray analysis. Childs Nerv Syst. 2009; 25: 13–20. 10.1007/s00381-008-0701-x 18818933

[85] Corcoran C, Rani S, O'Driscoll L. miR-34a is an intracellular and exosomal predictive biomarker for response to docetaxel with clinical relevance to prostate cancer progression. Prostate. 2014; 74: 1320–1334. 10.1002/pros.22848 25053345

[86] Ohdaira H, Nakagawa H, Yoshida K. Profiling of molecular pathways regulated by microRNA 601. Comput Biol Chem. 2009; 33: 429–433. 10.1016/j.compbiolchem.2009.09.003 19889580

[87] Veuger SJ, Hunter JE, Durkacz BW. Ionizing radiation-induced NF-kappaB activation requires PARP-1 function to confer radioresistance. Oncogene. 2009; 28: 832–842. 10.1038/onc.2008.439 19060926 PMC2642763

[88] Magne N, Toillon RA, Bottero V, Didelot C, Houtte PV, Gerard JP, et al NF-kappaB modulation and ionizing radiation: mechanisms and future directions for cancer treatment. Cancer Lett. 2006; 231: 158–168. 16399220 10.1016/j.canlet.2005.01.022

[89] Fan Y, Mao R, Yang J. NF-kappaB and STAT3 signaling pathways collaboratively link inflammation to cancer. Protein Cell. 2013; 4: 176–185. 10.1007/s13238-013-2084-3 23483479 PMC4875500

