# Supplementary material for: A Novel MiRNA-Based Predictive Model for Biochemical Failure Following Post-Prostatectomy Salvage Radiation Therapy
Source: PLoS One. 2015 Mar 11;10(3):e0118745. doi: 10.1371/journal.pone.0118745 (PMC4356539; doi:10.1371/journal.pone.0118745)
Supplement: S1 Fig — Patients were divided into high and low risk groups dichotomized by the median risk score. (DOCX) [file pone.0118745.s001.docx]

Figure S1. Kaplan-Meier plot estimates and AUC values of miRNA-based predictive salvage RT model without patients treated with hormonal therapy.


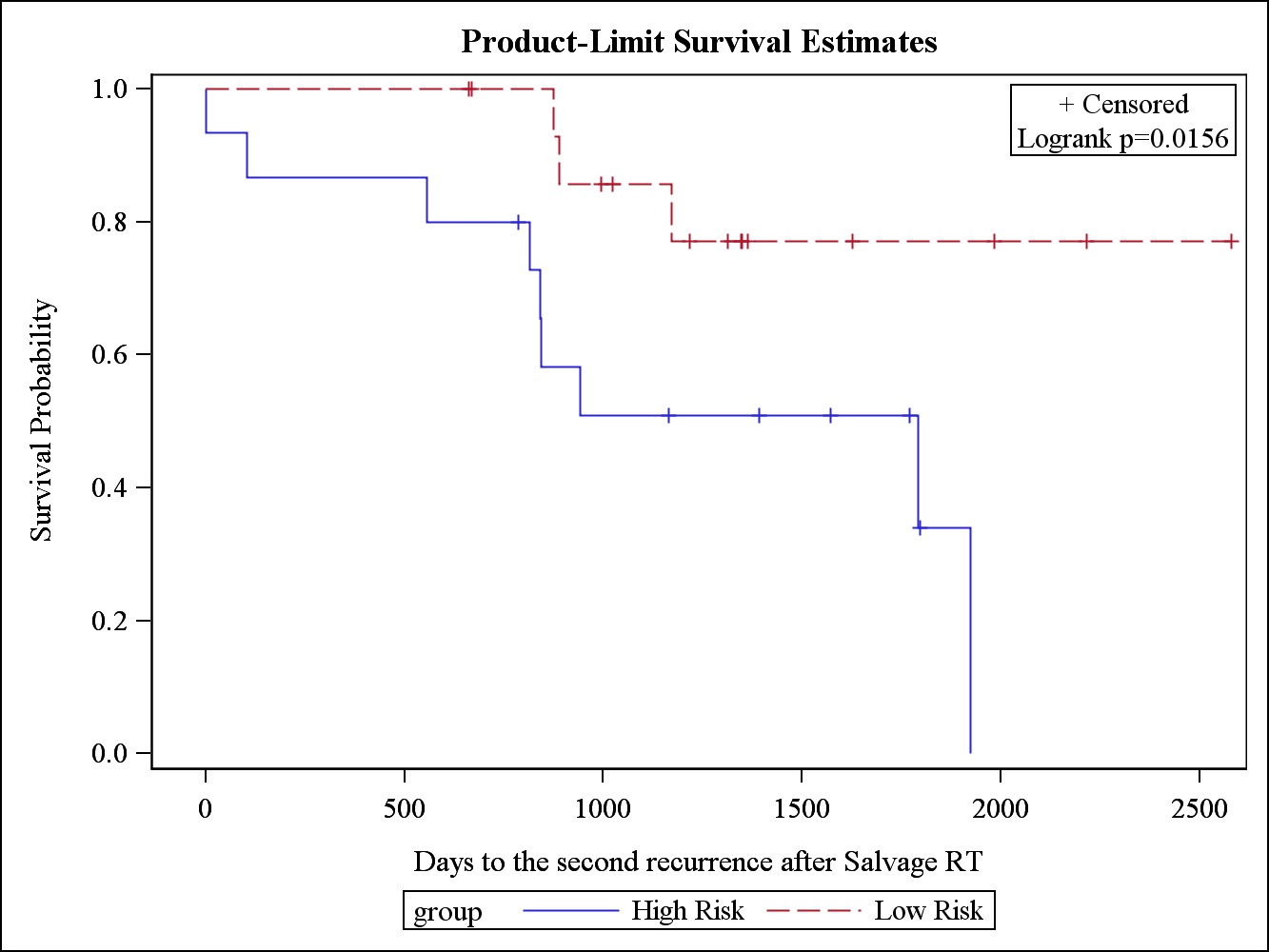


| Model | AUC (NNE Method) |
| --- | --- |
| Gleason+Lymph+miR601+miR4516 | 0.83 |
| Gleason+Lymph+miR601 | 0.79 |
| Gleason+Lymph+ miR4516 | 0.76 |
| Gleason+Lymph | 0.6 |
| miR601 | 0.76 |
| miR4516 | 0.71 |
| miR601+miR4516 | 0.77 |

Patients (n=31) were divided into high and low risk groups dichotomized by the median risk score.
