# Supplementary material for: A Novel MiRNA-Based Predictive Model for Biochemical Failure Following Post-Prostatectomy Salvage Radiation Therapy
Source: PLoS One. 2015 Mar 11;10(3):e0118745. doi: 10.1371/journal.pone.0118745 (PMC4356539; doi:10.1371/journal.pone.0118745)
Supplement: S1 Table — p-values were calculated using a repeated measured ANOVA model. (DOCX) [file pone.0118745.s002.docx]

| **miR_ID**  Table S1. miRNA comparisons between early vs late recurrence via ANOVA analysis. | **Ratio (Late: Early)** | **p-value** | **Mean (Late)** | **Mean (Early)** |
| --- | --- | --- | --- | --- |
| hsa-miR-145-5p | 0.32202 | 0.0007 | 1630.94 | 5064.73 |
| hsa-miR-221-3p | 0.42762 | 0.0001 | 58.83 | 137.58 |
| hsa-miR-27b-3p | 0.43656 | 0.0007 | 252.18 | 577.66 |
| hsa-miR-200a-3p | 0.45447 | 0.0026 | 109.13 | 240.12 |
| hsa-miR-143-3p | 0.4547 | 0.0031 | 2009.27 | 4418.93 |
| hsa-miR-200b-3p | 0.47031 | 0.0108 | 218.29 | 464.15 |
| hsa-miR-26a-5p | 0.49082 | 0.0108 | 710.78 | 1448.16 |
| hsa-miR-29c-3p | 0.49275 | 0.0017 | 363.69 | 738.07 |
| hsa-miR-23b-3p | 0.49425 | 0.0041 | 373.89 | 756.47 |
| hsa-miR-24-3p | 0.51956 | 0.0019 | 95.16 | 183.16 |
| hsa-let-7e-5p | 0.53013 | 0.0006 | 182.99 | 345.18 |
| hsa-miR-130a-3p | 0.53557 | 0.0028 | 185.47 | 346.3 |
| hsa-miR-30c-5p | 0.54826 | 0.0008 | 54.97 | 100.26 |
| hsa-let-7f-5p | 0.55346 | 0.0015 | 346.13 | 625.4 |
| hsa-miR-195-5p | 0.55717 | 0.002 | 101.2 | 181.64 |
| hsa-miR-199a-3p?miR-199b- | 0.56099 | 0.0098 | 346.44 | 617.55 |
| hsa-miR-98 | 0.56358 | 0.0007 | 90.94 | 161.37 |
| hsa-miR-720 | 0.56623 | 0.0049 | 9822.96 | 17347.88 |
| hsa-miR-376a-3p | 0.56675 | 0.0008 | 75.02 | 132.38 |
| hsa-miR-361-5p | 0.5668 | 0.0036 | 120.3 | 212.24 |
| hsa-let-7a-5p | 0.56943 | 0.0015 | 5104.76 | 8964.63 |
| hsa-let-7c | 0.5733 | 0.0017 | 759.01 | 1323.94 |
| hsa-miR-23a-3p | 0.57904 | 0.0087 | 649.3 | 1121.34 |
| hsa-miR-1 | 0.58131 | 0.0148 | 63.43 | 109.11 |
| hsa-miR-29b-3p | 0.58301 | 0.009 | 1144.22 | 1962.61 |
| hsa-miR-30b-5p | 0.59435 | 0.0202 | 457.48 | 769.71 |
| hsa-miR-125b-5p | 0.60235 | 0.0076 | 4021.05 | 6675.64 |
| hsa-let-7b-5p | 0.60465 | 0.0015 | 2390.02 | 3952.71 |
| hsa-miR-22-3p | 0.60552 | 0.0034 | 259.6 | 428.72 |
| hsa-miR-100-5p | 0.60641 | 0.0067 | 429.42 | 708.13 |
| hsa-miR-133a | 0.61408 | 0.0294 | 32.54 | 52.98 |
| hsa-miR-10b-5p | 0.61835 | 0.008 | 45.37 | 73.37 |
| hsa-miR-186-5p | 0.61856 | <.0001 | 49.96 | 80.78 |
| hsa-miR-34a-5p | 0.61913 | 0.0007 | 113.16 | 182.77 |
| hsa-miR-99a-5p | 0.62682 | 0.0164 | 841.28 | 1342.14 |
| hsa-miR-497-5p | 0.62691 | 0.0137 | 81.88 | 130.61 |
| hsa-miR-301b | 0.628 | 0.0053 | 27.76 | 44.21 |
| hsa-let-7g-5p | 0.62865 | 0.012 | 1335.33 | 2124.12 |
| hsa-miR-374b-5p | 0.63133 | 0.0028 | 111.47 | 176.56 |
| hsa-miR-125a-5p | 0.63288 | 0.0025 | 101.97 | 161.11 |
| hsa-miR-222-3p | 0.63736 | 0.015 | 191.35 | 300.23 |
| hsa-miR-199a-5p | 0.63879 | 0.0082 | 130.97 | 205.04 |
| hsa-miR-324-5p | 0.64374 | <.0001 | 57.02 | 88.57 |
| hsa-miR-16-5p | 0.64478 | 0.0266 | 377.17 | 584.96 |
| hsa-miR-15a-5p | 0.64586 | 0.0112 | 256.56 | 397.23 |
| hsa-miR-429 | 0.6493 | 0.0489 | 60.01 | 92.42 |
| hsa-let-7d-5p | 0.65561 | 0.0014 | 166.14 | 253.42 |
| hsa-miR-29a-3p | 0.65694 | 0.0251 | 283.02 | 430.82 |
| hsa-miR-342-3p | 0.65812 | 0.028 | 192.38 | 292.32 |
| hsa-miR-141-3p | 0.65813 | 0.0285 | 545.9 | 829.48 |
| hsa-miR-541-3p | 1.51627 | 0.0057 | 26.21 | 17.29 |
| hsa-miR-568 | 1.5289 | 0.0054 | 36.92 | 24.15 |
| hsa-miR-342-5p | 1.9266 | 0.0091 | 19.29 | 10.01 |
| hsa-miR-137 | 2.69857 | 0.0111 | 233.38 | 86.48 |

Tumor-only miRNA expression comparisons between patients with early (≤36 months) and late (>36 months) biochemical recurrence. p-values were generated using a repeated measured ANOVA model.
