# Supplementary material for: A Novel MiRNA-Based Predictive Model for Biochemical Failure Following Post-Prostatectomy Salvage Radiation Therapy
Source: PLoS One. 2015 Mar 11;10(3):e0118745. doi: 10.1371/journal.pone.0118745 (PMC4356539; doi:10.1371/journal.pone.0118745)
Supplement: S2 Table — Patients were divided into two groups by the median miRNA expression and a univariate log-rank test was performed to correlate expression with time to first biochemical recurrence. Significant p-value < 0.05. (DOCX) [file pone.0118745.s003.docx]

| **miR_ID**  Table S2. miRNAs that predict time to first recurrence via log-rank analysis. | **p-value (log-rank)** |
| --- | --- |
| hsa-miR-107 | 0.0001 |
| hsa-miR-576-3p | 0.0001 |
| hsa-let-7f-5p | 0.0003 |
| hsa-miR-1286 | 0.0003 |
| hsa-miR-486-3p | 0.0003 |
| hsa-miR-568 | 0.0005 |
| hsa-miR-106b-5p | 0.0006 |
| hsa-miR-890 | 0.0006 |
| hsa-miR-15a-5p | 0.0007 |
| hsa-miR-365a-3p | 0.0007 |
| hsa-miR-450b-5p | 0.0007 |
| hsa-miR-423-5p | 0.0008 |
| hsa-miR-572 | 0.0008 |
| hsa-miR-29c-3p | 0.0009 |
| hsa-miR-28-5p | 0.001 |
| hsa-miR-302a-3p | 0.0011 |
| hsa-miR-548aa | 0.0011 |
| hsa-miR-324-5p | 0.0014 |
| hsa-miR-18b-5p | 0.0015 |
| hsa-miR-1260b | 0.0016 |
| hsa-miR-141-3p | 0.0017 |
| hsa-miR-922 | 0.0019 |
| hsa-miR-148a-3p | 0.0021 |
| hsa-miR-148b-3p | 0.0022 |
| hsa-miR-98 | 0.0023 |
| hsa-miR-193a-5p | 0.0024 |
| hsa-miR-191-5p | 0.0025 |
| hsa-miR-548ak | 0.0027 |
| hsa-miR-1908 | 0.0029 |
| hsa-miR-548d-5p | 0.0033 |
| hsa-miR-375 | 0.0034 |
| hsa-miR-660-5p | 0.0034 |
| hsa-miR-194-5p | 0.0036 |
| hsa-miR-483-3p | 0.0037 |
| hsa-miR-1257 | 0.0038 |
| hsa-miR-4454 | 0.0038 |
| hsa-let-7a-5p | 0.0041 |
| hsa-miR-638 | 0.0041 |
| hsa-miR-30b-5p | 0.0043 |
| hsa-miR-30c-5p | 0.0044 |
| hsa-miR-106a-5p?miR-17-5p | 0.0045 |
| hsa-let-7e-5p | 0.0046 |
| hsa-miR-1276 | 0.0047 |
| hsa-miR-516a-3p | 0.0047 |
| hsa-miR-30d-5p | 0.0049 |
| hsa-miR-320a | 0.0049 |
| hsa-miR-216a | 0.0059 |
| hsa-miR-1323 | 0.0063 |
| hsa-miR-450b-3p | 0.0069 |
| hsa-miR-3136-5p | 0.0074 |
| hsa-let-7g-5p | 0.0075 |
| hsa-miR-93-5p | 0.0075 |
| hsa-miR-885-5p | 0.0082 |
| hsa-miR-1206 | 0.0085 |
| hsa-miR-421 | 0.0085 |
| hsa-miR-1200 | 0.0091 |
| hsa-miR-548am-3p | 0.0107 |
| hsa-miR-34c-3p | 0.0111 |
| hsa-miR-3934 | 0.0112 |
| hsa-miR-497-5p | 0.0122 |
| hsa-miR-1205 | 0.0123 |
| hsa-miR-15b-5p | 0.0123 |
| hsa-miR-508-5p | 0.0127 |
| hsa-miR-409-3p | 0.0128 |
| hsa-miR-376a-3p | 0.0134 |
| hsa-miR-762 | 0.0138 |
| hsa-miR-195-5p | 0.0141 |
| hsa-miR-654-5p | 0.0151 |
| hsa-miR-130a-3p | 0.0153 |
| hsa-miR-1290 | 0.0154 |
| hsa-miR-1288 | 0.0157 |
| hsa-miR-613 | 0.0163 |
| hsa-miR-1279 | 0.0168 |
| hsa-miR-376c | 0.017 |
| hsa-miR-135a-5p | 0.0177 |
| hsa-miR-23b-3p | 0.0178 |
| hsa-miR-1915-3p | 0.0182 |
| hsa-miR-27b-3p | 0.0187 |
| hsa-miR-362-5p | 0.0195 |
| hsa-miR-4448 | 0.0198 |
| hsa-miR-196a-5p | 0.0199 |
| hsa-miR-541-3p | 0.0203 |
| hsa-miR-548l | 0.0207 |
| hsa-miR-570-3p | 0.0214 |
| hsa-miR-767-3p | 0.0214 |
| hsa-let-7d-5p | 0.0215 |
| hsa-miR-92a-3p | 0.0219 |
| hsa-miR-24-3p | 0.0224 |
| hsa-miR-221-3p | 0.0225 |
| hsa-miR-3168 | 0.0225 |
| hsa-miR-199a-3p?miR-199b- | 0.0237 |
| hsa-miR-4508 | 0.0241 |
| hsa-miR-374b-5p | 0.0251 |
| hsa-miR-548t-5p | 0.0251 |
| hsa-miR-3180 | 0.0252 |
| hsa-miR-495 | 0.0279 |
| hsa-miR-19b-3p | 0.0286 |
| hsa-miR-1180 | 0.0291 |
| hsa-miR-3196 | 0.0304 |
| hsa-miR-566 | 0.0325 |
| hsa-miR-524-3p | 0.0331 |
| hsa-miR-429 | 0.0332 |
| hsa-miR-145-5p | 0.0361 |
| hsa-let-7i-5p | 0.0366 |
| hsa-miR-29b-3p | 0.0374 |
| hsa-miR-137 | 0.0377 |
| hsa-miR-149-5p | 0.0378 |
| hsa-miR-891b | 0.0382 |
| hsa-miR-514b-5p | 0.0394 |
| hsa-miR-549 | 0.0394 |
| hsa-miR-513a-3p | 0.0403 |
| hsa-miR-548z | 0.041 |
| hsa-miR-511 | 0.0423 |
| hsa-miR-99b-5p | 0.0424 |
| hsa-miR-548v | 0.0438 |
| hsa-miR-766-3p | 0.0439 |
| hsa-miR-1245b-5p | 0.0445 |
| hsa-miR-143-3p | 0.0465 |
| hsa-miR-877-5p | 0.0465 |
| hsa-miR-146b-5p | 0.0473 |
| hsa-miR-515-3p | 0.0484 |
| hsa-miR-1179 | 0.0485 |
| hsa-miR-20a-5p?miR-20b-5p | 0.0488 |

Tumor-only miRNA expression was used to predict time to first biochemical recurrence. Patients were divided into two groups by the median miRNA expression and a univariate log-rank test was performed to correlate expression with time to first biochemical recurrence. Significant p-value < 0.05.
