# Supplementary material for: A Novel MiRNA-Based Predictive Model for Biochemical Failure Following Post-Prostatectomy Salvage Radiation Therapy
Source: PLoS One. 2015 Mar 11;10(3):e0118745. doi: 10.1371/journal.pone.0118745 (PMC4356539; doi:10.1371/journal.pone.0118745)
Supplement: S3 Table — Hazards ratios were generated using a multivariate Cox regression analysis (initial PSA and Gleason score). Only miRNAs with a significant p-value (<0.05) are shown. (DOCX) [file pone.0118745.s004.docx]

Table S3. miRNAs that predict time to first biochemical recurrence post-radical prostatectomy (RP) via multivariate Cox regression analysis.

| **miR_ID** | **Hazard Ratio (High vs Low)** | **p-value** | **95% CI** |
| --- | --- | --- | --- |
| hsa-miR-107 | 9.61 | <.0001 | (3.47, 26.58) |
| hsa-miR-1915-3p | 8.0464 | 0.0002 | (2.65, 24.44) |
| hsa-miR-106b-5p | 5.5337 | 0.0008 | (2.03, 15.09) |
| hsa-miR-421 | 4.9287 | <.0001 | (2.28, 10.67) |
| hsa-let-7f-5p | 4.6887 | 0.0005 | (1.98, 11.12) |
| hsa-miR-18b-5p | 4.6492 | 0.001 | (1.87, 11.58) |
| hsa-miR-106a-5p?miR-17-5p | 4.565 | 0.0023 | (1.72, 12.12) |
| hsa-miR-15a-5p | 4.4285 | 0.0016 | (1.76, 11.13) |
| hsa-miR-191-5p | 4.1342 | 0.003 | (1.62, 10.54) |
| hsa-miR-1279 | 4.077 | 0.0015 | (1.71, 9.73) |
| hsa-miR-29c-3p | 3.8189 | 0.0014 | (1.68, 8.68) |
| hsa-miR-4454 | 3.8179 | 0.0037 | (1.55, 9.42) |
| hsa-miR-93-5p | 3.7934 | 0.0023 | (1.61, 8.93) |
| hsa-miR-376a-3p | 3.7741 | 0.0013 | (1.68, 8.47) |
| hsa-miR-423-5p | 3.7727 | 0.0014 | (1.67, 8.54) |
| hsa-miR-149-5p | 3.5734 | 0.0008 | (1.7, 7.52) |
| hsa-miR-20a-5p?miR-20b-5p | 3.4053 | 0.0074 | (1.39, 8.35) |
| hsa-miR-221-3p | 3.3326 | 0.0025 | (1.53, 7.27) |
| hsa-miR-141-3p | 3.1033 | 0.0113 | (1.29, 7.45) |
| hsa-miR-19b-3p | 3.077 | 0.0201 | (1.19, 7.94) |
| hsa-miR-877-5p | 3.0044 | 0.0149 | (1.24, 7.28) |
| hsa-miR-497-5p | 3.0009 | 0.0052 | (1.39, 6.49) |
| hsa-miR-148a-3p | 2.9478 | 0.0075 | (1.33, 6.51) |
| hsa-miR-27b-3p | 2.9186 | 0.007 | (1.34, 6.35) |
| hsa-miR-30c-5p | 2.916 | 0.0034 | (1.42, 5.97) |
| hsa-miR-28-5p | 2.9121 | 0.0025 | (1.46, 5.82) |
| hsa-let-7e-5p | 2.8575 | 0.0042 | (1.39, 5.86) |
| hsa-miR-376c | 2.8526 | 0.0051 | (1.37, 5.94) |
| hsa-miR-324-5p | 2.8459 | 0.0064 | (1.34, 6.04) |
| hsa-miR-98 | 2.8348 | 0.0031 | (1.42, 5.66) |
| hsa-miR-548aa | 2.8047 | 0.0133 | (1.24, 6.34) |
| hsa-miR-30d-5p | 2.7969 | 0.0085 | (1.3, 6.01) |
| hsa-miR-92a-3p | 2.7473 | 0.016 | (1.21, 6.25) |
| hsa-miR-549 | 2.7108 | 0.0164 | (1.2, 6.12) |
| hsa-let-7g-5p | 2.7026 | 0.0053 | (1.34, 5.44) |
| hsa-miR-570-3p | 2.6726 | 0.0123 | (1.24, 5.77) |
| hsa-miR-365a-3p | 2.5484 | 0.0241 | (1.13, 5.75) |
| hsa-miR-146b-5p | 2.5192 | 0.0225 | (1.14, 5.57) |
| hsa-miR-23b-3p | 2.4507 | 0.0129 | (1.21, 4.97) |
| hsa-miR-194-5p | 2.4328 | 0.0246 | (1.12, 5.28) |
| hsa-let-7a-5p | 2.4173 | 0.0116 | (1.22, 4.8) |
| hsa-miR-483-3p | 2.4078 | 0.0429 | (1.03, 5.64) |
| hsa-miR-130a-3p | 2.3785 | 0.0139 | (1.19, 4.74) |
| hsa-miR-193a-5p | 2.3649 | 0.0259 | (1.11, 5.04) |
| hsa-miR-3196 | 2.3296 | 0.0196 | (1.15, 4.74) |
| hsa-miR-195-5p | 2.3243 | 0.0153 | (1.18, 4.6) |
| hsa-miR-548v | 2.3194 | 0.0484 | (1.01, 5.35) |
| hsa-miR-1260b | 2.2893 | 0.0388 | (1.04, 5.02) |
| hsa-miR-374b-5p | 2.2845 | 0.0228 | (1.12, 4.65) |
| hsa-miR-24-3p | 2.2661 | 0.0204 | (1.14, 4.52) |
| hsa-miR-1290 | 2.2529 | 0.032 | (1.07, 4.73) |
| hsa-miR-199a-3p?miR-199b- | 2.2506 | 0.0237 | (1.11, 4.54) |
| hsa-miR-135a-5p | 2.2052 | 0.0232 | (1.11, 4.36) |
| hsa-let-7d-5p | 2.121 | 0.0379 | (1.04, 4.31) |
| hsa-miR-3136-5p | 0.4684 | 0.0344 | (0.23, 0.95) |
| hsa-miR-891b | 0.4554 | 0.0418 | (0.21, 0.97) |
| hsa-miR-762 | 0.4527 | 0.0277 | (0.22, 0.92) |
| hsa-miR-1276 | 0.4493 | 0.0347 | (0.21, 0.94) |
| hsa-miR-1288 | 0.4302 | 0.015 | (0.22, 0.85) |
| hsa-miR-1205 | 0.4298 | 0.0173 | (0.21, 0.86) |
| hsa-miR-3180 | 0.4203 | 0.0266 | (0.2, 0.9) |
| hsa-miR-613 | 0.4178 | 0.0252 | (0.19, 0.9) |
| hsa-miR-1257 | 0.4124 | 0.0323 | (0.18, 0.93) |
| hsa-miR-1323 | 0.4113 | 0.0329 | (0.18, 0.93) |
| hsa-miR-548l | 0.408 | 0.0206 | (0.19, 0.87) |
| hsa-miR-922 | 0.4068 | 0.0257 | (0.18, 0.9) |
| hsa-miR-508-5p | 0.3865 | 0.0117 | (0.18, 0.81) |
| hsa-miR-516a-3p | 0.3848 | 0.0219 | (0.17, 0.87) |
| hsa-miR-34c-3p | 0.3818 | 0.0076 | (0.19, 0.77) |
| hsa-miR-3934 | 0.3657 | 0.0095 | (0.17, 0.78) |
| hsa-miR-548t-5p | 0.3582 | 0.0067 | (0.17, 0.75) |
| hsa-miR-196a-5p | 0.3462 | 0.0051 | (0.16, 0.73) |
| hsa-miR-890 | 0.3455 | 0.0123 | (0.15, 0.79) |
| hsa-miR-137 | 0.3454 | 0.0039 | (0.17, 0.71) |
| hsa-miR-486-3p | 0.3411 | 0.0075 | (0.16, 0.75) |
| hsa-miR-511 | 0.3411 | 0.0168 | (0.14, 0.82) |
| hsa-miR-885-5p | 0.3404 | 0.0034 | (0.17, 0.7) |
| hsa-miR-495 | 0.3343 | 0.0028 | (0.16, 0.69) |
| hsa-miR-638 | 0.3327 | 0.0029 | (0.16, 0.69) |
| hsa-miR-766-3p | 0.3316 | 0.0029 | (0.16, 0.69) |
| hsa-miR-320a | 0.3305 | 0.0085 | (0.14, 0.75) |
| hsa-miR-1200 | 0.3237 | 0.0039 | (0.15, 0.7) |
| hsa-miR-566 | 0.3135 | 0.0084 | (0.13, 0.74) |
| hsa-miR-450b-5p | 0.3114 | 0.0064 | (0.13, 0.72) |
| hsa-miR-4508 | 0.3077 | 0.0024 | (0.14, 0.66) |
| hsa-miR-548ak | 0.2636 | 0.0011 | (0.12, 0.59) |
| hsa-miR-1908 | 0.2623 | 0.0027 | (0.11, 0.63) |
| hsa-miR-409-3p | 0.2597 | 0.0017 | (0.11, 0.6) |
| hsa-miR-1286 | 0.2488 | 0.0016 | (0.1, 0.59) |
| hsa-miR-450b-3p | 0.2468 | 0.0027 | (0.1, 0.61) |
| hsa-miR-654-5p | 0.2446 | 0.0003 | (0.11, 0.53) |
| hsa-miR-576-3p | 0.209 | 0.0004 | (0.09, 0.5) |
| hsa-miR-216a | 0.1914 | 0.0001 | (0.08, 0.45) |
| hsa-miR-568 | 0.18 | 0.0007 | (0.07, 0.49) |
| hsa-miR-302a-3p | 0.1646 | 0.0002 | (0.06, 0.42) |
| hsa-miR-572 | 0.1507 | <.0001 | (0.06, 0.35) |
| hsa-miR-1180 | 0.1279 | 0.0002 | (0.04, 0.38) |

miRNAs that predict time to failure post-RP (first recurrence) in salvage radiation patients. Hazards ratios were generated using a multivariate Cox regression analysis (initial PSA and Gleason score). Only miRNAs with a significant p-value (<0.05) are shown.
