# Supplementary material for: A Novel MiRNA-Based Predictive Model for Biochemical Failure Following Post-Prostatectomy Salvage Radiation Therapy
Source: PLoS One. 2015 Mar 11;10(3):e0118745. doi: 10.1371/journal.pone.0118745 (PMC4356539; doi:10.1371/journal.pone.0118745)
Supplement: S4 Table — Hazard ratios were generated using a multivariate Cox regression analysis (D’Amico, categorical Stephensen, or continuous Stephensen). Only miRNAs with a significant p-value (<0.05) are shown. (DOCX) [file pone.0118745.s005.docx]

| Table S4. miRNAs that predict biochemical recurrence post-radical prostatectomy (RP) via multivariate Cox-regression analysis. | **D’Amico nomogram** | | | **Stephensen nomogram (categorical)** | | | **Stephensen nomogram (continuous)** | | |
| --- | --- | --- | --- | --- | --- | --- | --- | --- | --- |
| **miR_ID** | **Hazard Ratio** | **p-value** | **Confidence Interval** | **Hazard Ratio** | **p-value** | **Confidence Interval** | **Hazard Ratio** | **p-value** | **Confidence Interval** |
| hsa-miR-107 | 3.69 | 0.0002 | (1.9, 7.3) | 3.42 | 0.0005 | (1.7, 6.9) | 3.37 | 0.0006 | (1.7, 6.7) |
| hsa-miR-98 | 3.49 | 0.0003 | (1.8, 6.9) | 2.37 | 0.0086 | (1.2, 4.5) | 2.44 | 0.0059 | (1.3, 4.6) |
| hsa-let-7a-5p | 3.24 | 0.0006 | (1.7, 6.4) | 2.18 | 0.0188 | (1.1, 4.2) | 2.23 | 0.0156 | (1.2, 4.3) |
| hsa-miR-324-5p | 3.03 | 0.0008 | (1.6, 5.8) | 3.08 | 0.0007 | (1.6, 5.9) | 2.92 | 0.0011 | (1.5, 5.6) |
| hsa-miR-106b-5p | 3.02 | 0.0031 | (1.5, 6.3) | 2.95 | 0.0050 | (1.4, 6.3) | 3.04 | 0.0040 | (1.4, 6.5) |
| hsa-let-7f-5p | 3.01 | 0.0013 | (1.5, 5.9) | 3.03 | 0.0017 | (1.5, 6.1) | 3.13 | 0.0011 | (1.6, 6.2) |
| hsa-let-7e-5p | 3.00 | 0.0009 | (1.6, 5.7) | 2.22 | 0.0140 | (1.2, 4.2) | 2.27 | 0.0106 | (1.2, 4.3) |
| hsa-miR-28-5p | 2.97 | 0.0011 | (1.5, 5.7) | 2.50 | 0.0054 | (1.3, 4.8) | 2.51 | 0.0058 | (1.3, 4.8) |
| hsa-miR-29c-3p | 2.94 | 0.0014 | (1.5, 5.7) | 3.04 | 0.0014 | (1.5, 6) | 2.86 | 0.0023 | (1.5, 5.6) |
| hsa-let-7g-5p | 2.94 | 0.0016 | (1.5, 5.7) | 2.20 | 0.0162 | (1.2, 4.2) | 2.22 | 0.0136 | (1.2, 4.2) |
| hsa-miR-148a-3p | 2.90 | 0.0032 | (1.4, 5.9) | 2.69 | 0.0071 | (1.3, 5.5) | 2.88 | 0.0036 | (1.4, 5.9) |
| hsa-miR-18b-5p | 2.78 | 0.0025 | (1.4, 5.4) | 4.23 | 0.0001 | (2.1, 8.7) | 4.02 | 0.0002 | (1.9, 8.4) |
| hsa-miR-15a-5p | 2.76 | 0.0030 | (1.4, 5.4) | 2.88 | 0.0026 | (1.4, 5.7) | 2.91 | 0.0022 | (1.5, 5.8) |
| hsa-miR-106a-5p + miR-17-5p | 2.72 | 0.0070 | (1.3, 5.6) | 2.79 | 0.0065 | (1.3, 5.8) | 2.69 | 0.0084 | (1.3, 5.6) |
| hsa-miR-365a-3p | 2.70 | 0.0055 | (1.3, 5.4) | 2.93 | 0.0027 | (1.5, 5.9) | 3.14 | 0.0013 | (1.6, 6.3) |
| hsa-miR-194-5p | 2.68 | 0.0091 | (1.3, 5.6) | 2.49 | 0.0226 | (1.1, 5.4) | 2.52 | 0.0177 | (1.2, 5.4) |
| hsa-miR-4454 | 2.67 | 0.0061 | (1.3, 5.4) | 2.73 | 0.0057 | (1.3, 5.6) | 2.70 | 0.0059 | (1.3, 5.5) |
| hsa-miR-191-5p | 2.67 | 0.0040 | (1.4, 5.2) | 2.55 | 0.0079 | (1.3, 5.1) | 2.65 | 0.0051 | (1.3, 5.2) |
| hsa-miR-141-3p | 2.64 | 0.0070 | (1.3, 5.3) | 2.68 | 0.0071 | (1.3, 5.5) | 2.74 | 0.0055 | (1.3, 5.6) |
| hsa-miR-548aa | 2.62 | 0.0048 | (1.3, 5.1) | 2.86 | 0.0063 | (1.3, 6.1) | 2.62 | 0.0069 | (1.3, 5.3) |
| hsa-miR-497-5p | 2.61 | 0.0035 | (1.4, 5) | 2.20 | 0.0146 | (1.2, 4.1) | 2.12 | 0.0185 | (1.1, 4) |
| hsa-miR-30c-5p | 2.60 | 0.0035 | (1.4, 4.9) | 2.89 | 0.0014 | (1.5, 5.5) | 2.68 | 0.0025 | (1.4, 5.1) |
| hsa-miR-4448 | 2.58 | 0.0052 | (1.3, 5) | 2.60 | 0.0052 | (1.3, 5.1) | 2.35 | 0.0107 | (1.2, 4.5) |
| hsa-miR-548v | 2.56 | 0.0074 | (1.3, 5.1) | 2.29 | 0.0158 | (1.2, 4.5) | 2.38 | 0.0129 | (1.2, 4.7) |
| hsa-miR-148b-3p | 2.52 | 0.0087 | (1.3, 5) | 2.70 | 0.0063 | (1.3, 5.5) | 2.61 | 0.0079 | (1.3, 5.3) |
| hsa-miR-1260b | 2.51 | 0.0072 | (1.3, 4.9) | 2.49 | 0.0158 | (1.2, 5.2) | 2.49 | 0.0109 | (1.2, 5) |
| hsa-miR-375 | 2.50 | 0.0093 | (1.3, 5) | 2.22 | 0.0380 | (1, 4.7) | 2.33 | 0.0262 | (1.1, 4.9) |
| hsa-miR-221-3p | 2.50 | 0.0052 | (1.3, 4.8) | 2.63 | 0.0036 | (1.4, 5) | 2.65 | 0.0041 | (1.4, 5.2) |
| hsa-miR-423-5p | 2.50 | 0.0101 | (1.2, 5) | 2.65 | 0.0073 | (1.3, 5.4) | 2.77 | 0.0044 | (1.4, 5.6) |
| hsa-miR-195-5p | 2.46 | 0.0050 | (1.3, 4.6) | 2.06 | 0.0251 | (1.1, 3.9) | 2.04 | 0.0242 | (1.1, 3.8) |
| hsa-miR-27b-3p | 2.42 | 0.0067 | (1.3, 4.6) | 2.13 | 0.0190 | (1.1, 4) | 2.03 | 0.0260 | (1.1, 3.8) |
| hsa-miR-483-3p | 2.42 | 0.0143 | (1.2, 4.9) | 2.33 | 0.0278 | (1.1, 5) | 2.40 | 0.0191 | (1.2, 5) |
| hsa-miR-193a-5p | 2.40 | 0.0101 | (1.2, 4.7) | 3.49 | 0.0006 | (1.7, 7.1) | 3.77 | 0.0004 | (1.8, 7.9) |
| hsa-miR-660-5p | 2.38 | 0.0104 | (1.2, 4.6) | 3.39 | 0.0010 | (1.6, 7) | 2.92 | 0.0020 | (1.5, 5.7) |
| hsa-miR-374b-5p | 2.37 | 0.0073 | (1.3, 4.5) | 1.96 | 0.0365 | (1, 3.7) | 1.93 | 0.0370 | (1, 3.6) |
| hsa-miR-1206 | 2.36 | 0.0145 | (1.2, 4.7) | 2.29 | 0.0234 | (1.1, 4.7) | 2.33 | 0.0184 | (1.2, 4.7) |
| hsa-miR-30b-5p | 2.32 | 0.0169 | (1.2, 4.6) | 3.64 | 0.0008 | (1.7, 7.7) | 3.03 | 0.0021 | (1.5, 6.1) |
| hsa-let-7d-5p | 2.31 | 0.0089 | (1.2, 4.3) | 1.91 | 0.0438 | (1, 3.6) | 1.95 | 0.0354 | (1, 3.6) |
| hsa-miR-135a-5p | 2.30 | 0.0120 | (1.2, 4.4) | 2.03 | 0.0281 | (1.1, 3.8) | 2.15 | 0.0165 | (1.2, 4) |
| hsa-miR-24-3p | 2.30 | 0.0095 | (1.2, 4.3) | 2.05 | 0.0238 | (1.1, 3.8) | 1.98 | 0.0307 | (1.1, 3.7) |
| hsa-miR-199a-3p + miR-199b | 2.28 | 0.0101 | (1.2, 4.3) | 1.93 | 0.0400 | (1, 3.6) | 1.94 | 0.0366 | (1, 3.6) |
| hsa-miR-130a-3p | 2.15 | 0.0164 | (1.2, 4) | 2.03 | 0.0288 | (1.1, 3.8) | 2.04 | 0.0254 | (1.1, 3.8) |
| hsa-miR-1915-3p | 2.15 | 0.0200 | (1.1, 4.1) | 2.46 | 0.0091 | (1.3, 4.8) | 2.12 | 0.0237 | (1.1, 4) |
| hsa-miR-99b-5p | 2.15 | 0.0198 | (1.1, 4.1) | 2.06 | 0.0305 | (1.1, 4) | 1.88 | 0.0479 | (1, 3.5) |
| hsa-miR-93-5p | 2.11 | 0.0315 | (1.1, 4.2) | 2.68 | 0.0049 | (1.3, 5.3) | 2.45 | 0.0099 | (1.2, 4.8) |
| hsa-miR-30d-5p | 2.10 | 0.0229 | (1.1, 4) | 2.35 | 0.0119 | (1.2, 4.6) | 2.36 | 0.0080 | (1.3, 4.4) |
| hsa-miR-15b-5p | 2.05 | 0.0347 | (1.1, 4) | 2.38 | 0.0132 | (1.2, 4.7) | 2.25 | 0.0178 | (1.2, 4.4) |
| hsa-miR-23b-3p | 2.00 | 0.0303 | (1.1, 3.7) | 2.00 | 0.0318 | (1.1, 3.8) | 2.01 | 0.0286 | (1.1, 3.7) |
| hsa-miR-421 | 1.95 | 0.0393 | (1, 3.7) | 1.98 | 0.0393 | (1, 3.8) | 2.06 | 0.0265 | (1.1, 3.9) |
| hsa-miR-145-5p | 1.88 | 0.0462 | (1, 3.5) | 2.49 | 0.0059 | (1.3, 4.8) | 2.49 | 0.0069 | (1.3, 4.8) |
| hsa-miR-548t-5p | 0.53 | 0.0449 | (0.3, 1) | 0.35 | 0.0026 | (0.2, 0.7) | 0.39 | 0.0049 | (0.2, 0.8) |
| hsa-miR-1180 | 0.52 | 0.0464 | (0.3, 1) | 0.35 | 0.0028 | (0.2, 0.7) | 0.36 | 0.0044 | (0.2, 0.7) |
| hsa-miR-515-3p | 0.52 | 0.0471 | (0.3, 1) | 0.51 | 0.0437 | (0.3, 1) | 0.50 | 0.0334 | (0.3, 0.9) |
| hsa-miR-508-5p | 0.51 | 0.0365 | (0.3, 1) | 0.31 | 0.0012 | (0.2, 0.6) | 0.35 | 0.0021 | (0.2, 0.7) |
| hsa-miR-513a-3p | 0.51 | 0.0428 | (0.3, 1) | 0.39 | 0.0064 | (0.2, 0.8) | 0.45 | 0.0169 | (0.2, 0.9) |
| hsa-miR-566 | 0.51 | 0.0310 | (0.3, 0.9) | 0.42 | 0.0130 | (0.2, 0.8) | 0.50 | 0.0282 | (0.3, 0.9) |
| hsa-miR-613 | 0.50 | 0.0363 | (0.3, 1) | 0.39 | 0.0059 | (0.2, 0.8) | 0.41 | 0.0088 | (0.2, 0.8) |
| hsa-miR-1200 | 0.50 | 0.0353 | (0.3, 1) | 0.38 | 0.0041 | (0.2, 0.7) | 0.35 | 0.0027 | (0.2, 0.7) |
| hsa-miR-762 | 0.49 | 0.0323 | (0.3, 0.9) | 0.30 | 0.0010 | (0.2, 0.6) | 0.33 | 0.0021 | (0.2, 0.7) |
| hsa-miR-450b-3p | 0.49 | 0.0324 | (0.2, 0.9) | 0.42 | 0.0113 | (0.2, 0.8) | 0.44 | 0.0149 | (0.2, 0.9) |
| hsa-miR-1205 | 0.48 | 0.0210 | (0.3, 0.9) | 0.42 | 0.0139 | (0.2, 0.8) | 0.45 | 0.0136 | (0.2, 0.9) |
| hsa-miR-3168 | 0.48 | 0.0212 | (0.3, 0.9) | 0.38 | 0.0059 | (0.2, 0.8) | 0.40 | 0.0064 | (0.2, 0.8) |
| hsa-miR-548d-5p | 0.47 | 0.0471 | (0.2, 1) | 0.45 | 0.0327 | (0.2, 0.9) | 0.41 | 0.0189 | (0.2, 0.9) |
| hsa-miR-320a | 0.47 | 0.0271 | (0.2, 0.9) | 0.41 | 0.0095 | (0.2, 0.8) | 0.41 | 0.0100 | (0.2, 0.8) |
| hsa-miR-3136-5p | 0.47 | 0.0231 | (0.2, 0.9) | 0.47 | 0.0266 | (0.2, 0.9) | 0.45 | 0.0173 | (0.2, 0.9) |
| hsa-miR-3934 | 0.47 | 0.0166 | (0.2, 0.9) | 0.41 | 0.0058 | (0.2, 0.8) | 0.37 | 0.0030 | (0.2, 0.7) |
| hsa-miR-409-3p | 0.45 | 0.0190 | (0.2, 0.9) | 0.41 | 0.0100 | (0.2, 0.8) | 0.34 | 0.0032 | (0.2, 0.7) |
| hsa-miR-516a-3p | 0.45 | 0.0216 | (0.2, 0.9) | 0.40 | 0.0089 | (0.2, 0.8) | 0.40 | 0.0074 | (0.2, 0.8) |
| hsa-miR-1288 | 0.45 | 0.0128 | (0.2, 0.8) | 0.50 | 0.0340 | (0.3, 0.9) | 0.49 | 0.0282 | (0.3, 0.9) |
| hsa-miR-541-3p | 0.44 | 0.0108 | (0.2, 0.8) | 0.41 | 0.0060 | (0.2, 0.8) | 0.44 | 0.0101 | (0.2, 0.8) |
| hsa-miR-1257 | 0.44 | 0.0250 | (0.2, 0.9) | 0.27 | 0.0005 | (0.1, 0.6) | 0.25 | 0.0005 | (0.1, 0.5) |
| hsa-miR-548ak | 0.44 | 0.0139 | (0.2, 0.8) | 0.26 | 0.0003 | (0.1, 0.5) | 0.27 | 0.0004 | (0.1, 0.6) |
| hsa-miR-1323 | 0.44 | 0.0171 | (0.2, 0.9) | 0.45 | 0.0200 | (0.2, 0.9) | 0.45 | 0.0232 | (0.2, 0.9) |
| hsa-miR-216a | 0.41 | 0.0076 | (0.2, 0.8) | 0.24 | 0.0002 | (0.1, 0.5) | 0.34 | 0.0018 | (0.2, 0.7) |
| hsa-miR-922 | 0.41 | 0.0097 | (0.2, 0.8) | 0.30 | 0.0006 | (0.1, 0.6) | 0.32 | 0.0011 | (0.2, 0.6) |
| hsa-miR-302a-3p | 0.41 | 0.0067 | (0.2, 0.8) | 0.36 | 0.0053 | (0.2, 0.7) | 0.39 | 0.0041 | (0.2, 0.7) |
| hsa-miR-1276 | 0.40 | 0.0110 | (0.2, 0.8) | 0.35 | 0.0045 | (0.2, 0.7) | 0.35 | 0.0033 | (0.2, 0.7) |
| hsa-miR-34c-3p | 0.40 | 0.0046 | (0.2, 0.8) | 0.36 | 0.0028 | (0.2, 0.7) | 0.40 | 0.0046 | (0.2, 0.8) |
| hsa-miR-572 | 0.39 | 0.0048 | (0.2, 0.8) | 0.25 | 0.0003 | (0.1, 0.5) | 0.35 | 0.0014 | (0.2, 0.7) |
| hsa-miR-1908 | 0.36 | 0.0029 | (0.2, 0.7) | 0.40 | 0.0098 | (0.2, 0.8) | 0.39 | 0.0067 | (0.2, 0.8) |
| hsa-miR-568 | 0.35 | 0.0026 | (0.2, 0.7) | 0.25 | 0.0001 | (0.1, 0.5) | 0.28 | 0.0003 | (0.1, 0.6) |
| hsa-miR-576-3p | 0.35 | 0.0028 | (0.2, 0.7) | 0.27 | 0.0002 | (0.1, 0.5) | 0.28 | 0.0002 | (0.1, 0.5) |
| hsa-miR-486-3p | 0.34 | 0.0020 | (0.2, 0.7) | 0.28 | 0.0004 | (0.1, 0.6) | 0.29 | 0.0004 | (0.1, 0.6) |
| hsa-miR-638 | 0.33 | 0.0010 | (0.2, 0.6) | 0.31 | 0.0005 | (0.2, 0.6) | 0.32 | 0.0007 | (0.2, 0.6) |
| hsa-miR-885-5p | 0.31 | 0.0007 | (0.2, 0.6) | 0.44 | 0.0122 | (0.2, 0.8) | 0.42 | 0.0070 | (0.2, 0.8) |
| hsa-miR-1286 | 0.31 | 0.0013 | (0.1, 0.6) | 0.33 | 0.0047 | (0.2, 0.7) | 0.30 | 0.0016 | (0.1, 0.6) |
| hsa-miR-450b-5p | 0.28 | 0.0005 | (0.1, 0.6) | 0.33 | 0.0019 | (0.2, 0.7) | 0.31 | 0.0010 | (0.2, 0.6) |
| hsa-miR-890 | 0.25 | 0.0001 | (0.1, 0.5) | 0.27 | 0.0006 | (0.1, 0.6) | 0.34 | 0.0025 | (0.2, 0.7) |

miRNAs that predict time to failure post-RP (first recurrence) in salvage radiation patients. Hazard ratios were generated using a multivariate Cox regression analysis (D’Amico, categorical Stephensen, or continuous Stephensen). Only miRNAs with a significant p-value (<0.05) are shown.
