# Supplementary material for: A Novel MiRNA-Based Predictive Model for Biochemical Failure Following Post-Prostatectomy Salvage Radiation Therapy
Source: PLoS One. 2015 Mar 11;10(3):e0118745. doi: 10.1371/journal.pone.0118745 (PMC4356539; doi:10.1371/journal.pone.0118745)
Supplement: S5 Table — p-values were generated using a repeated measured ANOVA. (DOCX) [file pone.0118745.s006.docx]

| **miR_ID** | **Ratio (Recurrence : Non-recurrence)** | **p-value** | **Mean (Non-recurrence)** | **Mean (Recurrence)** |
| --- | --- | --- | --- | --- |
| hsa-miR-4443 | 0.63 | 0.008 | 213.5 | 133.89 |
| hsa-miR-626 | 0.65 | 0.002 | 38.89 | 25.45 |
| hsa-miR-1202 | 0.67 | <.0001 | 33.95 | 22.64 |
| hsa-miR-10b-5p | 1.61 | 0.009 | 47.5 | 76.6 |

Table S5. miRNAs that predict biochemical recurrence post-salvage radiation therapy (RT) via ANOVA.

Tumor-only miRNA expression comparisons between patients with biochemical recurrence post-salvage RT (second biochemical recurrence) versus no recurrence post-salvage radiation therapy. p-values were generated using a repeated measured ANOVA.
