# Supplementary material for: A Novel MiRNA-Based Predictive Model for Biochemical Failure Following Post-Prostatectomy Salvage Radiation Therapy
Source: PLoS One. 2015 Mar 11;10(3):e0118745. doi: 10.1371/journal.pone.0118745 (PMC4356539; doi:10.1371/journal.pone.0118745)
Supplement: S6 Table — Patients were divided into two groups by the median miRNA expression and a univariate log-rank test was performed to correlate expression with second biochemical recurrence. Significant p-value < 0.05. (DOCX) [file pone.0118745.s007.docx]

| **miR_ID** | **p-value (log-rank)** |
| --- | --- |
| hsa-miR-628-3p | 0.0028 |
| hsa-miR-924 | 0.0039 |
| hsa-miR-626 | 0.0056 |
| hsa-miR-1202 | 0.0096 |
| hsa-miR-563 | 0.0234 |
| hsa-miR-598 | 0.0249 |
| hsa-miR-30d-5p | 0.0293 |
| hsa-miR-491-5p | 0.0298 |
| hsa-miR-4516 | 0.0326 |
| hsa-miR-601 | 0.0327 |
| hsa-miR-508-3p | 0.0356 |
| hsa-miR-576-5p | 0.0358 |
| hsa-miR-320e | 0.0394 |
| hsa-miR-134 | 0.0401 |
| hsa-miR-548an | 0.043 |
| hsa-miR-1303 | 0.0443 |
| hsa-miR-551a | 0.0449 |
| hsa-miR-761 | 0.0464 |
| hsa-miR-1244 | 0.0469 |
| hsa-miR-96-5p | 0.0471 |
| hsa-miR-658 | 0.0476 |
| hsa-miR-1193 | 0.0481 |
| hsa-miR-597 | 0.0482 |
| hsa-miR-1913 | 0.0483 |

Table S6. miRNAs that predict biochemical recurrence post-salvage radiation therapy (RT) via log-rank analysis.

Tumor-only miRNA expression was used to predict biochemical recurrence post-salvage RT (second biochemical recurrence). Patients were divided into two groups by the median miRNA expression and a univariate log-rank test was performed to correlate expression with second biochemical recurrence. Significant p-value < 0.05.
