# Supplementary material for: A Novel MiRNA-Based Predictive Model for Biochemical Failure Following Post-Prostatectomy Salvage Radiation Therapy
Source: PLoS One. 2015 Mar 11;10(3):e0118745. doi: 10.1371/journal.pone.0118745 (PMC4356539; doi:10.1371/journal.pone.0118745)
Supplement: S7 Table — All targets of both miR-1193 and miR-626 are listed with scores according to both TargetScan and microRNA.org. (DOCX) [file pone.0118745.s008.docx]

Table S7. Putative Targets of novel miRNAs, miR-1193 and miR-626, associated with biochemical recurrence post-salvage RT (second biochemical recurrence).

| **miR-1193 target gene** | **TargetScan total context+ score** | **miR-1193 target gene** | **microRNA.org mirSVR score** | **miR-626 target gene** | **TargetScan total context+ score** | **miR-626 target gene** | **microRNA.org mirSVR score** |
| --- | --- | --- | --- | --- | --- | --- | --- |
| FAM169B | -2.92 | FAM169B | -3.53 | DPY19L3 | -0.76 | DPY19L3 | -2.55 |
| GSG1L | -1.05 | WDR67 | -2.83 | PAPOLB | -0.76 | CWC27 | -2.51 |
| FTSJD1 | -0.71 | TXNDC2 | -2.81 | GPATCH4 | -0.67 | AZI2 | -2.34 |
| GPM6A | -0.59 | USP15 | -2.65 | SPOPL | -0.65 | RAB1A | -2.09 |
| GRIPAP1 | -0.58 | TMCO4 | -2.51 | BACH2 | -0.64 | C2orf55 | -2.02 |
| PEG3 | -0.57 | C14orf145 | -2.4 | CENPP | -0.62 | RBM39 | -1.99 |
| LPPR5 | -0.48 | OOSP1 | -2.11 | TMPRSS11BNL | -0.58 | KIAA1704 | -1.98 |
| IL36B | -0.48 | PTK2 | -2.04 | HDX | -0.55 | C12orf59 | -1.93 |
| SMG6 | -0.47 | FTSJD1 | -2.02 | ACSL3 | -0.52 | PLK1S1 | -1.68 |
| MID2 | -0.45 | GSG1L | -1.96 | RBM39 | -0.51 | ZNF660 | -1.76 |
| PPP1CB | -0.44 | UBE2CBP | -1.78 | ARFGAP3 | -0.51 | STK33 | -1.7 |
| FCRL1 | -0.41 | TRIM27 | -1.6 | NFYA | -0.51 | ERBB2IP | -1.68 |
| CHD9 | -0.39 | ERMAP | -1.65 | ATP5C1 | -0.5 | PTBP2 | -0.14 |
| PPM1E | -0.38 | VAT1L | -1.65 | DYRK4 | -0.5 | SLC2A1 | -1.64 |
| ARID2 | -0.38 | NOL10 | -1.61 | LPIN2 | -0.49 | TFAP2D | -1.6 |
| CDK5R1 | -0.38 | GNG13 | -1.58 | LRRC4 | -0.49 | DTNA | -0.05 |
| TLK2 | -0.38 | SMG6 | -1.56 | HCN4 | -0.47 | KIAA1598 | -1.57 |
| TAF7L | -0.38 | CTTNBP2NL | -0.01 | MYEF2 | -0.46 | ARHGEF6 | -1.54 |
| ATP10B | -0.36 | KDELR2 | -1.5 | SOX9 | -0.46 | UBXN7 | -1.54 |
| MTSS1L | -0.36 | CDC14A | -1.48 | CAMKK2 | -0.45 | DGKB | -1.52 |
| PDE1B | -0.36 | NKAIN2 | -1.45 | TMCC1 | -0.43 | ZNF398 | -1.51 |
| GALNT2 | -0.3 | LSM12 | -1.45 | RNF6 | -0.42 | SFRS18 | -1.5 |
| ZSCAN20 | -0.3 | BCAM | -1.43 | IGFBP5 | -0.42 | ARID4B | -1.49 |
| LASP1 | -0.29 | MGC11082 | -1.42 | INPP5A | -0.41 | PAPOLB | -1.48 |
| LAMC2 | -0.29 | DGKH | -1.39 | BCL11A | -0.4 | CNTNAP3B | -1.48 |
| DCAF8 | -0.29 | NMI | -1.35 | TRIM3 | -0.4 | EYA1 | -1.45 |
| GLCE | -0.28 | FAM71F1 | -1.33 | MRTO4 | -0.4 | TLL1 | -1.44 |
| KPNA1 | -0.28 | TOP2A | -1.33 | SELT | -0.4 | ANKRD19 | -1.41 |
| DAAM2 | -0.27 | SCN8A | -1.32 | CLCC1 | -0.4 | GPD2 | -1.4 |
| SORT1 | -0.27 | ASZ1 | -1.32 | FOXC1 | -0.39 | TRAPPC2 | -1.39 |
| UBR1 | -0.27 | KL | -1.31 | SLC18A2 | -0.39 | KNDC1 | -1.35 |
| PTEN | -0.26 | EXOSC3 | -1.31 | FREM2 | -0.39 | KCNH8 | -1.35 |
| OBFC1 | -0.26 | TRAFD1 | -1.3 | CASQ2 | -0.39 | ANKS3 | -1.34 |
| BCORL1 | -0.26 | RDH10 | -1.29 | APOBEC2 | -0.38 | ATP5C1 | -1.33 |
| SPEG | -0.26 | NBEAL1 | -1.29 | MAGI2 | -0.38 | ACER2 | -1.33 |
| RDH10 | -0.25 | PDK1 | -1.28 | IGF1 | -0.38 | MGAT2 | -1.32 |
| NCK2 | -0.25 | CHD2 | -1.28 | ARMC10 | -0.37 | CREBL2 | -1.32 |
| FBXL5 | -0.25 | SMAD4 | -1.28 | BCAT1 | -0.37 | GNG2 | -0.44 |
| EFNB1 | -0.24 | GRIPAP1 | -1.28 | NAA15 | -0.36 | FIP1L1 | -1.17 |
| CALCR | -0.23 | SUCLG2 | -1.28 | SMO | -0.35 | STAG3L4 | -1.31 |
| RNF144A | -0.23 | VRK2 | -1.26 | DVL2 | -0.35 | SLTM | -1.31 |
| DCX | -0.22 | ITGA1 | -1.26 | WDR55 | -0.35 | HNRNPA1 | -1.3 |
| SYT9 | -0.22 | MSTN | -1.26 | CACNA1I | -0.34 | TRIM23 | -1.2 |
| CPLX2 | -0.22 | LARS | -1.26 | MPRIP | -0.34 | RNF6 | -1.29 |
| TXNL4B | -0.22 | PCF11 | -1.25 | MTDH | -0.33 | ZIM3 | -1.29 |
| KDELR2 | -0.2 | DAAM2 | -1.25 | CHRAC1 | -0.33 | MS4A4E | -1.2 |
| RGL1 | -0.2 | CADM2 | -1.25 | SLAIN1 | -0.32 | ATP5S | -1.29 |
| DCUN1D5 | -0.2 | SPATA6 | -1.25 | RBMS1 | -0.32 | PAIP2 | -1.29 |
| CD93 | -0.19 | RNASET2 | -1.25 | TNIK | -0.31 | DYRK4 | -1.29 |
| KIAA1755 | -0.19 | LRRC1 | -1.25 | FAM84A | -0.31 | NCKAP5 | -1.29 |
| CCDC85C | -0.18 |  |  | DAB2IP | -0.3 |  |  |
| KCNA1 | -0.18 |  |  | GLIS3 | -0.3 |  |  |
| UBN2 | -0.18 |  |  | CLDN18 | -0.29 |  |  |
| KSR2 | -0.18 |  |  | CTPS | -0.29 |  |  |
| TBL2 | -0.18 |  |  | CNR1 | -0.29 |  |  |
| BAI3 | -0.17 |  |  | KIAA1671 | -0.28 |  |  |
| GANC | -0.17 |  |  | MBNL1 | -0.28 |  |  |
| SMURF1 | -0.17 |  |  | PDAP1 | -0.28 |  |  |
| ATL2 | -0.16 |  |  | B4GALT4 | -0.28 |  |  |
| OTUD5 | -0.16 |  |  | 3-Mar | -0.28 |  |  |
| SPTBN4 | -0.16 |  |  | SETD7 | -0.27 |  |  |
| RPS6KA3 | -0.15 |  |  | MAP2K7 | -0.27 |  |  |
| TM9SF3 | -0.14 |  |  | MXD3 | -0.27 |  |  |
| PIP4K2C | -0.14 |  |  | RAB1A | -0.27 |  |  |
| SPIN1 | -0.13 |  |  | UBL3 | -0.26 |  |  |
| PTGIR | -0.13 |  |  | STARD9 | -0.26 |  |  |
| ELK1 | -0.13 |  |  | ATP5F1 | -0.26 |  |  |
| FOXH1 | -0.13 |  |  | ZNF609 | -0.26 |  |  |
| LRP5L | -0.13 |  |  | MGRN1 | -0.26 |  |  |
| CDH24 | -0.13 |  |  | ELL | -0.25 |  |  |
| GJA5 | -0.13 |  |  | PRKG1 | -0.25 |  |  |
| HELZ | -0.12 |  |  | HDAC8 | -0.24 |  |  |
| RAP1GDS1 | -0.12 |  |  | GLE1 | -0.24 |  |  |
| RIMS1 | -0.12 |  |  | MEIS2 | -0.24 |  |  |
| AGPAT6 | -0.12 |  |  | RYBP | -0.24 |  |  |
| VASH1 | -0.11 |  |  | C3orf70 | -0.24 |  |  |
| TPM4 | -0.11 |  |  | ODZ4 | -0.24 |  |  |
| VGLL3 | -0.1 |  |  | NEO1 | -0.24 |  |  |
| RNF8 | -0.1 |  |  | LMOD3 | -0.23 |  |  |
| LARP4B | -0.1 |  |  | ZDHHC22 | -0.23 |  |  |
| SHANK3 | -0.1 |  |  | WBSCR17 | -0.23 |  |  |
| SIN3A | -0.09 |  |  | GNG2 | -0.23 |  |  |
| ZNF385A | -0.09 |  |  | GIT2 | -0.22 |  |  |
| TAOK1 | -0.08 |  |  | NUDT11 | -0.22 |  |  |
| ENY2 | -0.07 |  |  | LIF | -0.22 |  |  |
| ANKRD52 | -0.07 |  |  | SGPL1 | -0.22 |  |  |
| MTDH | -0.07 |  |  | MAP1B | -0.21 |  |  |
| DIAPH1 | -0.07 |  |  | RRP15 | -0.21 |  |  |
| TUB | -0.07 |  |  | NPAS3 | -0.21 |  |  |
| PTGS1 | -0.07 |  |  | CRNN | -0.21 |  |  |
| PCGF3 | -0.06 |  |  | PPM1E | -0.21 |  |  |
| HLF | -0.05 |  |  | PRCD | -0.21 |  |  |
| RND2 | -0.04 |  |  | KCNE1L | -0.21 |  |  |
| ITGB3 | -0.04 |  |  | JPH1 | -0.21 |  |  |
| ETS1 | -0.04 |  |  | COPS2 | -0.2 |  |  |
| RBBP4 | > -0.02 |  |  | ADAM19 | -0.2 |  |  |
| FBN1 | N/A |  |  | SCRT2 | -0.2 |  |  |
| SPATA6 | N/A |  |  | SDC1 | -0.2 |  |  |
|  |  |  |  | ZC3H11A | -0.2 |  |  |
|  |  |  |  | HTR4 | -0.2 |  |  |
|  |  |  |  | GPHN | -0.19 |  |  |
|  |  |  |  | USP28 | -0.19 |  |  |
|  |  |  |  | PANK1 | -0.19 |  |  |
|  |  |  |  | SERBP1 | -0.19 |  |  |
|  |  |  |  | PHF23 | -0.19 |  |  |
|  |  |  |  | NFAM1 | -0.18 |  |  |
|  |  |  |  | SMARCD1 | -0.18 |  |  |
|  |  |  |  | PPP3R1 | -0.18 |  |  |
|  |  |  |  | SLC9A9 | -0.17 |  |  |
|  |  |  |  | TLK2 | -0.17 |  |  |
|  |  |  |  | LMX1B | -0.17 |  |  |
|  |  |  |  | LAS1L | -0.17 |  |  |
|  |  |  |  | ACER2 | -0.17 |  |  |
|  |  |  |  | PPAP2B | -0.17 |  |  |
|  |  |  |  | C15orf32 | -0.17 |  |  |
|  |  |  |  | GRB10 | -0.17 |  |  |
|  |  |  |  | DMXL1 | -0.17 |  |  |
|  |  |  |  | LMOD1 | -0.17 |  |  |
|  |  |  |  | FOXO1 | -0.16 |  |  |
|  |  |  |  | MCTS1 | -0.16 |  |  |
|  |  |  |  | UBR5 | -0.16 |  |  |
|  |  |  |  | ZNF526 | -0.16 |  |  |
|  |  |  |  | ETV6 | -0.15 |  |  |
|  |  |  |  | MECP2 | -0.15 |  |  |
|  |  |  |  | SEMA6A | -0.15 |  |  |
|  |  |  |  | OSBPL3 | -0.15 |  |  |
|  |  |  |  | TNNI3K | -0.14 |  |  |
|  |  |  |  | FPGT-TNNI3K | -0.14 |  |  |
|  |  |  |  | TYW3 | -0.14 |  |  |
|  |  |  |  | EXOC5 | -0.14 |  |  |
|  |  |  |  | CORO1C | -0.14 |  |  |
|  |  |  |  | EIF4E3 | -0.13 |  |  |
|  |  |  |  | BCR | -0.13 |  |  |
|  |  |  |  | AFF4 | -0.13 |  |  |
|  |  |  |  | SHROOM3 | -0.13 |  |  |
|  |  |  |  | ESYT1 | -0.13 |  |  |
|  |  |  |  | MKL2 | -0.12 |  |  |
|  |  |  |  | SYPL2 | -0.12 |  |  |
|  |  |  |  | RASAL2 | -0.12 |  |  |
|  |  |  |  | C1orf144 | -0.12 |  |  |
|  |  |  |  | UBE2QL1 | -0.12 |  |  |
|  |  |  |  | ZBTB47 | -0.12 |  |  |
|  |  |  |  | SEC61A2 | -0.12 |  |  |
|  |  |  |  | ZFAT | -0.12 |  |  |
|  |  |  |  | PDSS2 | -0.12 |  |  |
|  |  |  |  | CARM1 | -0.12 |  |  |
|  |  |  |  | KIAA0040 | -0.11 |  |  |
|  |  |  |  | ZBTB38 | -0.11 |  |  |
|  |  |  |  | SCN4B | -0.11 |  |  |
|  |  |  |  | ABHD2 | -0.1 |  |  |
|  |  |  |  | DMRTB1 | -0.1 |  |  |
|  |  |  |  | LARP4B | -0.1 |  |  |
|  |  |  |  | XRRA1 | -0.1 |  |  |
|  |  |  |  | FOXO3 | -0.09 |  |  |
|  |  |  |  | AKAP5 | -0.09 |  |  |
|  |  |  |  | PYGO2 | -0.09 |  |  |
|  |  |  |  | SOX1 | -0.09 |  |  |
|  |  |  |  | CSRNP3 | -0.09 |  |  |
|  |  |  |  | ARFGAP2 | -0.09 |  |  |
|  |  |  |  | DIXDC1 | -0.09 |  |  |
|  |  |  |  | KY | -0.08 |  |  |
|  |  |  |  | VAPB | -0.08 |  |  |
|  |  |  |  | SOX11 | -0.08 |  |  |
|  |  |  |  | JHDM1D | -0.08 |  |  |
|  |  |  |  | MDGA2 | -0.08 |  |  |
|  |  |  |  | PTMS | -0.08 |  |  |
|  |  |  |  | CTIF | -0.08 |  |  |
|  |  |  |  | CA12 | -0.07 |  |  |
|  |  |  |  | LPP | -0.07 |  |  |
|  |  |  |  | TUB | -0.07 |  |  |
|  |  |  |  | LOC400950 | -0.07 |  |  |
|  |  |  |  | KCNN3 | -0.07 |  |  |
|  |  |  |  | MFSD4 | -0.07 |  |  |
|  |  |  |  | MOCS1 | -0.07 |  |  |
|  |  |  |  | RIMKLA | -0.07 |  |  |
|  |  |  |  | FBXO41 | -0.06 |  |  |
|  |  |  |  | WNK3 | -0.06 |  |  |
|  |  |  |  | DLG2 | -0.05 |  |  |
|  |  |  |  | CREB5 | -0.05 |  |  |
|  |  |  |  | RAD23A | -0.05 |  |  |
|  |  |  |  | AKT3 | -0.04 |  |  |
|  |  |  |  | PRPF4B | -0.04 |  |  |
|  |  |  |  | LOX | -0.04 |  |  |
|  |  |  |  | BMP8B | -0.03 |  |  |
|  |  |  |  | PODXL | > -0.03 |  |  |
|  |  |  |  | DNM3 | > -0.03 |  |  |
|  |  |  |  | HAP1 | -0.02 |  |  |
|  |  |  |  | NFIA | > -0.02 |  |  |
|  |  |  |  | CBX7 | > -0.02 |  |  |
|  |  |  |  | MEX3A | > -0.02 |  |  |
|  |  |  |  | RUNX1T1 | -0.02 |  |  |

All targets of both miR-1193 and miR-626 are listed with scores according to both Targetscan and microRNA.org.
