# Supplementary material for: A Novel MiRNA-Based Predictive Model for Biochemical Failure Following Post-Prostatectomy Salvage Radiation Therapy
Source: PLoS One. 2015 Mar 11;10(3):e0118745. doi: 10.1371/journal.pone.0118745 (PMC4356539; doi:10.1371/journal.pone.0118745)
Supplement: S8 Table — All targets of both miR-601 and miR-4516 are listed with scores according to both TargetScan and microRNA.org. miR-4516 was not listed in microRNA.org. (DOCX) [file pone.0118745.s009.docx]

Table S8. Putative Targets of miRNAs, miR-601 and miR-4516, within our predictive model of biochemical recurrence post-salvage RT (second biochemical recurrence).

| **miR-601 target gene** | **TargetScan total context+ score** | **miR-601 target gene** | **microRNA.org miRSVR score** | **miR-4516 target gene** | **TargetScan total context+ score** |
| --- | --- | --- | --- | --- | --- |
| SGK494 | -0.96 | PMCHL1 | -1.82 | PHF8 | -0.54 |
| LHFPL2 | -0.73 | PMCHL2 | -1.76 | TRIM46 | -0.48 |
| PRKACB | -0.64 | CA2 | -1.67 | GRAMD2 | -0.45 |
| B3GNT9 | -0.53 | GOLGA8A | -1.54 | ST6GAL1 | -0.45 |
| EEA1 | -0.53 | NLGN1 | -1.54 | MTL5 | -0.43 |
| SNN | -0.51 | CDHR1 | -1.54 | PPP1R11 | -0.43 |
| CUL3 | -0.5 | BEYLA | -1.48 | CREB5 | -0.37 |
| SIRT1 | -0.46 | LRRCC1 | -0.43 | GAS2 | -0.35 |
| KCNJ12 | -0.45 | FMN2 | -1.44 | HCFC1 | -0.35 |
| TEC | -0.44 | TSPAN8 | -1.42 | TMEM199 | -0.35 |
| ISOC1 | -0.44 | ZBTB38 | -1.36 | KHNYN | -0.34 |
| FBLN7 | -0.43 | EFNA4 | -1.34 | PPP2R1A | -0.34 |
| GPX8 | -0.42 | RPL23AP82 | -1.34 | RNF10 | -0.34 |
| FBXO42 | -0.42 | JAK1 | -1.31 | ATOH8 | -0.33 |
| TROVE2 | -0.41 | SLC38A9 | -1.31 | IPP | -0.33 |
| PIK3R3 | -0.41 | TAF11 | -1.28 | SPOCK1 | -0.33 |
| ZBTB4 | -0.4 | CSRNP3 | -1.28 | EIF4B | -0.32 |
| NRG3 | -0.38 | SIRT1 | -1.27 | OPN1LW | -0.32 |
| TXNDC6 | -0.38 | POU2F2 | -1.27 | LRP4 | -0.31 |
| BCL2L2 | -0.36 | APOO | -1.27 | FXR2 | -0.3 |
| GLIPR2 | -0.35 | LOC151009 | -1.26 | HIF3A | -0.3 |
| RGS9BP | -0.35 | BCL2L2 | -1.26 | SDK2 | -0.3 |
| PRR14L | -0.32 | B3GNT9 | -1.26 | GPR173 | -0.29 |
| ANKRD6 | -0.32 | CCDC23 | -1.25 | NUMA1 | -0.29 |
| EIF4EBP3 | -0.32 | CDKAL1 | -1.25 | VAMP1 | -0.29 |
| ANKHD1-EIF4EBP3 | -0.32 | CCDC147 | -1.25 | ZNF202 | -0.28 |
| HLF | -0.32 | HLA-F | 1.25 | FAM110B | -0.27 |
| WDR87 | -0.3 | MFSD8 | -1.24 | FAM49A | -0.27 |
| LBH | -0.3 | LOC440894 | -1.24 | MARK2 | -0.27 |
| FAM60A | -0.29 | ALDH3A1 | -1.24 | MYO1E | -0.27 |
| PTPN11 | -0.28 | SNN | -1.23 | CDK2AP2 | -0.26 |
| TMEM132B | -0.28 | FGF20 | -1.23 | ZNF652 | -0.26 |
| PTP4A1 | -0.28 | C9orf150 | -1.23 | ITGA2B | -0.24 |
| ALG9 | -0.28 | EEF1E1 | -1.22 | RECQL5 | -0.24 |
| TSTD2 | -0.26 | FBXO42 | -1.22 | SHOX2 | -0.24 |
| PCDH11Y | -0.26 | RAB7L1 | -1.22 | ARHGAP30 | -0.22 |
| ZBTB38 | -0.25 | SGK494 | -1.22 | CCDC165 | -0.22 |
| MCMBP | -0.25 | TMCO5A | -1.21 | ETV4 | -0.22 |
| GPR12 | -0.25 | PACRGL | -1.21 | MR1 | -0.22 |
| UNC5C | -0.24 | ZBTB4 | -1.21 | PITX3 | -0.22 |
| CD109 | -0.24 | GRM3 | -1.2 | ALG9 | -0.21 |
| ST8SIA3 | -0.23 | GUF1 | -1.2 | BCAS3 | -0.21 |
| TRAF5 | -0.22 | SH3BGR | -1.2 | OLFML2B | -0.21 |
| AQP1 | -0.22 | FAM60A | -1.19 | PDE4A | -0.21 |
| C6orf35 | -0.22 | RASGRP3 | -1.19 | PPP2R5D | -0.21 |
| DYRK1A | -0.22 | PPP1R13B | -1.19 | RFX1 | -0.21 |
| PPP2R2D | -0.21 | CASP3 | -1.18 | SMG7 | -0.21 |
| LHX1 | -0.21 | TROVE2 | -1.18 | CYSLTR2 | -0.2 |
| YWHAZ | -0.2 | NRG3 | -1.18 | DHCR7 | -0.2 |
| JUP | -0.2 | DYRK1A | -1.18 | AAK1 | -0.19 |
| ZNF385A | -0.2 |  |  | B3GNT7 | -0.19 |
| ELAVL1 | -0.19 |  |  | CD2BP2 | -0.19 |
| AKAP2 | -0.19 |  |  | OSBPL7 | -0.19 |
| MGA | -0.19 |  |  | TADA3 | -0.19 |
| PALM2-AKAP2 | -0.19 |  |  | GPD1 | -0.18 |
| NFKBIZ | -0.18 |  |  | LIN28A | -0.18 |
| FOXG1 | -0.17 |  |  | STMN3 | -0.18 |
| MBNL1 | -0.17 |  |  | TEDDM1 | -0.18 |
| TAOK1 | -0.17 |  |  | ZNF853 | -0.18 |
| ZBTB44 | -0.17 |  |  | GNAI2 | -0.17 |
| PRRC2B | -0.16 |  |  | GRIK3 | -0.17 |
| KIAA0141 | -0.16 |  |  | NEK8 | -0.17 |
| SLC6A6 | -0.16 |  |  | NEUROD2 | -0.17 |
| PSMA5 | -0.15 |  |  | UBE2N | -0.17 |
| DUSP5 | -0.15 |  |  | PRKACA | -0.16 |
| ZNF710 | -0.15 |  |  | SLC25A29 | -0.16 |
| POU2F1 | -0.15 |  |  | EIF2C1 | -0.15 |
| RICTOR | -0.15 |  |  | SLC7A1 | -0.15 |
| KIAA1462 | -0.15 |  |  | CASC3 | -0.14 |
| C5orf51 | -0.14 |  |  | DDX17 | -0.14 |
| NRP2 | -0.14 |  |  | PALM | -0.14 |
| ZDHHC17 | -0.14 |  |  | TMOD1 | -0.14 |
| SEC61A1 | -0.14 |  |  | WDTC1 | -0.14 |
| FAM19A5 | -0.14 |  |  | ADD2 | -0.13 |
| CYTH3 | -0.13 |  |  | BRD3 | -0.13 |
| SH2B3 | -0.13 |  |  | G3BP2 | -0.13 |
| WIZ | -0.13 |  |  | PDPN | -0.13 |
| TSC1 | -0.13 |  |  | CACYBP | -0.12 |
| MIER1 | -0.13 |  |  | PEA15 | -0.12 |
| CLMN | -0.13 |  |  | ST8SIA2 | -0.12 |
| RBBP4 | -0.12 |  |  | TMEM151A | -0.12 |
| MATR3 | -0.12 |  |  | VANGL2 | -0.12 |
| IQSEC1 | -0.12 |  |  | AFF4 | -0.11 |
| ZFHX4 | -0.12 |  |  | C1orf95 | -0.11 |
| CDC73 | -0.12 |  |  | CHAD | -0.11 |
| SIRPA | -0.11 |  |  | HELZ | -0.11 |
| SOX11 | -0.11 |  |  | HMG20A | -0.11 |
| SH3TC2 | -0.11 |  |  | IGDCC3 | -0.11 |
| AAK1 | -0.11 |  |  | KIAA1045 | -0.11 |
| KCNK10 | -0.1 |  |  | BSN | -0.1 |
| EML1 | -0.1 |  |  | KLF3 | -0.1 |
| PURB | -0.09 |  |  | NLGN2 | -0.1 |
| FAM120C | -0.08 |  |  | RPS6KA3 | -0.1 |
| XIAP | -0.08 |  |  | TNS1 | -0.1 |
| INSIG1 | -0.07 |  |  | MNT | -0.09 |
| ETNK1 | -0.05 |  |  | APBA1 | -0.08 |
| NFAT5 | > -0.03 |  |  | SLC25A1 | -0.08 |
| ARRB1 | -0.01 |  |  | SP1 | -0.08 |
|  |  |  |  | NUP50 | -0.07 |
|  |  |  |  | PAG1 | -0.07 |
|  |  |  |  | PHF15 | -0.07 |
|  |  |  |  | SAMD4B | -0.07 |
|  |  |  |  | TLX1 | -0.07 |
|  |  |  |  | TSPAN5 | -0.07 |
|  |  |  |  | ZNF618 | -0.07 |
|  |  |  |  | CREB3L2 | -0.06 |
|  |  |  |  | FBXO45 | -0.06 |
|  |  |  |  | GGA1 | -0.06 |
|  |  |  |  | RAP1GDS1 | -0.06 |
|  |  |  |  | PDLIM2 | -0.05 |
|  |  |  |  | TRIOBP | -0.05 |
|  |  |  |  | GGCX | -0.04 |
|  |  |  |  | NPAS4 | -0.02 |

All targets of both miR-601 and miR-4516 are listed with scores according to both Targetscan and microRNA.org. miR-4516 was not listed in microRNA.org.
