# Supplementary material for: A Novel MiRNA-Based Predictive Model for Biochemical Failure Following Post-Prostatectomy Salvage Radiation Therapy
Source: PLoS One. 2015 Mar 11;10(3):e0118745. doi: 10.1371/journal.pone.0118745 (PMC4356539; doi:10.1371/journal.pone.0118745)
Supplement: S9 Table — Twelve genes with the lowest scores and highest probability of being targeted by miR-601 according to TargetScan and microRNA.org are listed with their known gene functions. (DOCX) [file pone.0118745.s010.docx]

Table S9. Top 12 putative gene targets and function for miR-601.

| **miR-601 Target Gene** | **TargetScan Total Context+ Score** | **miR-601 Target Gene** | **micorRNA.org miRSVR Score** | **Gene Function** |
| --- | --- | --- | --- | --- |
| B3GNT9 | -0.53 | B3GNT9 | -1.26 | Beta-1,3-N-acetylglucosaminyltransferase |
| BCL2L2 | -0.36 | BCL2L2 | -1.26 | Bcl2-like 2 protein 2; apoptosis pathway. |
| DYRK1A | -0.22 | DYRK1A | -1.18 | Dual specificity tyrosine-phosphorylation -regulated kinase 1A; roles in apoptotic resistance, proliferation, and migration. |
| FAM60A | -0.29 | FAM60A | -1.19 | Family with sequence similarity 60 member A; role in cell cycle pathway. |
| FBXO42 | -0.42 | FBXO42 | -1.22 | Just one F-Box and kelch domain-containing protein; protein ubiquitin ligase. |
| NRG3 | -0.38 | NRG3 | -1.18 | Neuroregulin 3; stimulates ERBB3 phosphorylation. |
| SGK494 | -0.96 | SGK494 | -1.22 | Sugen kinase 494; uncharacterized serine/threonine protein kinase. |
| SIRT1 | -0.46 | SIRT1 | -1.27 | Silent mating type information regulation 2 homolog; NAD-dependent deacetylase cell growth, immune response, DNA damage repair, hypoxia response, and energy homeostasis pathways. |
| SNN | -0.51 | SNN | -1.23 | Stannin protein |
| TROVE2 | -0.41 | TROVE2 | -1.18 | 60 kDa SS-A/Ro Rib nucleoprotein; RNA-binding protein. |
| ZBTB38 | -0.25 | ZBTB38 | -1.36 | Zinc finger and BTB domain-containing protein 38; zinc finger transcriptional repressor that binds methylated DNA; role in caspase-induced apoptosis. |
| ZBTB4 | -0.4 | ZBTB4 | -1.21 | Zinc finger and BTB domain containing 4; zinc finger transcriptional repressor that binds methylated DNA; role in cell cycle and apoptosis pathways. |

The twelve genes with the lowest scores and highest probability of being targeted by miR-601 according to Targetscan and microRNA.org are listed with their known gene functions.
