# Supplementary material for: A Novel MiRNA-Based Predictive Model for Biochemical Failure Following Post-Prostatectomy Salvage Radiation Therapy
Source: PLoS One. 2015 Mar 11;10(3):e0118745. doi: 10.1371/journal.pone.0118745 (PMC4356539; doi:10.1371/journal.pone.0118745)
Supplement: S10 Table — Ten genes with the lowest scores and highest probability of being targeted by miR-4516 according to TargetScan are listed with their known gene functions. (DOCX) [file pone.0118745.s011.docx]

| **miR-4516 Target Gene** | **TargetScan Total Context+ Score** | **Gene Function** |
| --- | --- | --- |
| PHF8 | -0.54 | PHD Finger Protein 8; histone lysine demethylase; involved in the carcinogenesis of tumors |
| TRIM46 | -0.48 | Tripartite Motif Containing 46; protein-coding gene |
| GRAMD2 | -0.45 | GRAM Domain-Containing Protein 2; protein-coding gene |
| ST6GAL1 | -0.45 | ST6 Beta-Galactosamide Alpha-2,6-Sialyltranferase 1; protein-coding gene for carbohydrate determinants and differentiation antigens; promotes tumorigenesis; regulator of the stem cell phenotype |
| MTL5 | -0.43 | Metallothionein-Like 5, Testis-Specific (Tesmin); role in the regulation of cell  growth and differentiation and are involved in spermatogenesis |
| PPP1R11 | -0.43 | Protein Phosphatase 1, Regulatory (Inhibitor) Subunit 11; inhibitor of protein phosphatase-1 |
| CREB5 | -0.37 | cAMP Responsive Element Binding Protein 5; CRE-dependent transcriptional activator; role in metastasis. |
| GAS2 | -0.35 | Growth Arrest-Specific 2; caspase-3 substrate; role in apoptosis |
| HCFC1 | -0.35 | Host Cell Factor C1; DNA and chromatin-binding protein; cell cycle and transcriptional regulation. |
| TMEM199 | -0.35 | Transmembrane Protein 199 |

Table S10. Top 10 putative gene targets and function for miR-4516.

The ten genes with the lowest scores and highest probability of being targeted by miR-4516 according to Targetscan are listed with their known gene functions.
