# Supplementary material for: A Novel MiRNA-Based Predictive Model for Biochemical Failure Following Post-Prostatectomy Salvage Radiation Therapy
Source: PLoS One. 2015 Mar 11;10(3):e0118745. doi: 10.1371/journal.pone.0118745 (PMC4356539; doi:10.1371/journal.pone.0118745)
Supplement: S11 Table — Displayed are top networks, diseases and disorders, and molecular and cellular functions. (DOCX) [file pone.0118745.s012.docx]

| Top Networks |  |  |
| --- | --- | --- |
| Associated Network Functions | **Score** |  |
| **Cancer, Gastrointestinal Disease, Respiratory Disease** | 41 |  |
| **Embryonic Development, Organismal Development, Cellular Development** | 25 |  |
| **Cell Signaling, Molecular Transport, Nucleic Acid Metabolism** | 18 |  |
| **Cell-To-Cell Signaling and Interaction, Digestive System Development and Function, Cell Signaling** | 16 |  |
| **Cellular Movement, Cell Morphology, Cellular Growth and Proliferation** | 16 |  |
| Top Diseases and Functions |  |  |
| Diseases and Disorders | **p-value** | **# Molecules** |
| **Organismal Injury and Abnormalities** | 6.37E-23 - 4.50E-02 | 45 |
| **Reproductive System Disease** | 6.37E-23 - 3.45E-02 | 34 |
| **Cancer** | 1.35E-21 - 4.89E-02 | 39 |
| **Inflammatory Disease** | 1.37E-19 - 2.96E-02 | 25 |
| **Inflammatory Response** | 1.37E-19 - 4.50E-02 | 22 |
| Molecular and Cellular Functions | **p-value** | **# Molecules** |
| **Cell Cycle** | 4.72E-12 - 4.87E-02 | 12 |
| **Cellular Movement** | 4.72E-12 - 3.45E-02 | 17 |
| **Cellular Development** | 3.33E-07 - 4.41E-02 | 21 |
| **Cellular Growth and Proliferation** | 3.33E-07 - 3.69E-02 | 19 |
| **DNA Replication, Recombination, and Repair** | 2.33E-04 - 1.30E-02 | 11 |

Table S11. Pathway analysis results for 88-miRNA signature.

Ingenuity Pathway Analysis (IPA) was performed on the 88-miRNA signature associated with time to first biochemical recurrence. Displayed are top networks, diseases and disorders, and molecular and cellular functions.
