# Supplementary material for: A Novel MiRNA-Based Predictive Model for Biochemical Failure Following Post-Prostatectomy Salvage Radiation Therapy
Source: PLoS One. 2015 Mar 11;10(3):e0118745. doi: 10.1371/journal.pone.0118745 (PMC4356539; doi:10.1371/journal.pone.0118745)
Supplement: S12 Table — Displayed are top networks and diseases and disorders. (DOCX) [file pone.0118745.s013.docx]

| Top Networks |  |  |
| --- | --- | --- |
| Associated Network Functions | **Score** |  |
| **Cancer, Cell Morphology, Cellular Assembly and Organization** | 27 |  |
| Top Diseases and Functions |  |  |
| Diseases and Disorders | **p-value** | **# Molecules** |
| **Organismal Injury and Abnormalities** | 3.24E-04 - 2.05E-02 | 4 |
| **Reproductive System Disease** | 3.24E-04 - 3.24E-04 | 2 |
| **Cancer** | 8.32E-03 - 8.32E-03 | 1 |
| **Hematological Disease** | 8.32E-03 - 8.32E-03 | 1 |
| **Immunological Disease** | 8.32E-03 - 8.32E-03 | 1 |

Table S12. Pathway analysis results for miRNAs in Predictive Salvage RT Model.

Ingenuity Pathway Analysis (IPA) was performed on the 9-miRNAs associated with biochemical recurrence post-salvage radiation therapy. Displayed are top networks and diseases and disorders.
